# Illuminating the COP1/SPA Ubiquitin Ligase: Fresh Insights Into Its Structure and Functions During Plant Photomorphogenesis

**DOI:** 10.3389/fpls.2021.662793

**Published:** 2021-03-24

**Authors:** Jathish Ponnu, Ute Hoecker

**Affiliations:** Institute for Plant Sciences, Cluster of Excellence on Plant Sciences (CEPLAS), Biocenter, University of Cologne, Cologne, Germany

**Keywords:** COP1, SPA, CUL4, C3D, CDD, DDB1, E3 ligase, ubiquitination

## Abstract

CONSTITUTIVE PHOTOMORPHOGENIC 1 functions as an E3 ubiquitin ligase in plants and animals. Discovered originally in *Arabidopsis thaliana*, COP1 acts in a complex with SPA proteins as a central repressor of light-mediated responses in plants. By ubiquitinating and promoting the degradation of several substrates, COP1/SPA regulates many aspects of plant growth, development and metabolism. In contrast to plants, human COP1 acts as a crucial regulator of tumorigenesis. In this review, we discuss the recent important findings in COP1/SPA research including a brief comparison between COP1 activity in plants and humans.

## Introduction

Plants are versatile organisms that coordinate growth and development by constantly sensing and responding to various internal and external signals. By integrating the information from multiple signals at the molecular level, plants orchestrate complex downstream functions that maximize their evolutionary fitness. Among the external signals that influence plant development, light plays a pivotal role. Besides supplying energy for photosynthesis, light functions as an important developmental cue that shapes the life of plants starting from seed germination to senescence. Plants perceive different wavelengths of light via specialized photoreceptors and appropriately change the gene expression patterns that result in photomorphogenesis or light-driven plant growth.

Photoreceptors accomplish a major part of their signaling functions via regulating the activities of CONSTITUTIVE PHOTOMORPHOGENIC 1 (COP1), a master regulator of light signaling. Discovered more than two decades ago in the model plant *Arabidopsis thaliana* (Arabidopsis), COP1 is among the first-known repressors of photomorphogenesis (Deng et al., 1991). COP1, a Really Interesting New Gene (RING)-finger E3 ubiquitin ligase, exists widely in eukaryotes including mammals. It polyubiquitinates and facilitates the proteasome-mediated degradation of numerous substrates (Wang et al., 1999; Yi and Deng, 2005; Lau and Deng, 2012; Han et al., 2020; Table 1). E3 ubiquitin ligases identify and associate with protein substrates, recruit the ubiquitin conjugating enzyme E2, and assist or directly involve in the transfer of ubiquitin molecules from E2 to the substrates (Mazzucotelli et al., 2006). In plants, the E3 ligase activity of COP1 depends on its interaction with SUPPRESSOR OF PHYA-105 (SPA) proteins (Hoecker and Quail, 2001; Seo et al., 2003; Laubinger et al., 2004; Zhu et al., 2008; Ordoñez-Herrera et al., 2015).

**TABLE 1:** Substrates of the Arabidopsis COP1/SPA E3 ligase.

**Substrate**	**AGI code**	**Identity**	**Interaction with**		**Functions in**	**References**
			**COP1**	**SPA**		
ABI1	AT4G26080	Protein phosphatase 2C	Yes	Nd	ABA signaling	Chen et al., 2020
AHG3	AT3G11410	Protein phosphatase 2C	Yes	Nd	ABA signaling	Chen et al., 2020
BBX1/CO	AT5G15840	B-box zinc finger protein	Yes	Yes	Flowering	Laubinger et al., 2006; Jang et al., 2008; Sarid-Krebs et al., 2015
BBX4/COL3	AT2G24790	B-box zinc finger protein	Yes	Nd	Photomorphogenesis	Heng et al., 2019
BBX10/COL12	AT3G21880	B-box zinc finger protein	Yes	Yes	Flowering	Ordoñez-Herrera et al., 2018
BBX20/BZS1	AT4G39070	B-box zinc finger protein	Yes	Nd	Photomorphogenesis, BR signaling	Fan et al., 2012
BBX21	AT1G75540	B-box zinc finger protein	Yes	Nd	Photomorphogenesis	Xu et al., 2016a
BBX22/STH3/LZF1	AT1G78600	B-box zinc finger protein	Yes	Nd	Photomorphogenesis	Datta et al., 2008; Chang et al., 2011
BBX24/STO	AT1G06040	B-box zinc finger protein	Yes	Nd	Photomorphogenesis	Holm et al., 2001; Yan et al., 2011; Jiang et al., 2012
BBX25/STH	AT2G31380	B-box zinc finger protein	Yes	Nd	Photomorphogenesis	Gangappa et al., 2013
BBX28	AT4G27310	B-box zinc finger protein	Yes	Nd	Photomorphogenesis	Lin et al., 2018
BBX29	AT5G54470	B-box zinc finger protein	Yes	Nd	Photomorphogenesis	Song Z. et al., 2020
BIT1	AT2G36890	MYB transcription factor	Yes	Nd	Photomorphogenesis	Hong et al., 2008
BZR1	AT1G75080	Transcription factor	Yes	Nd	BR signaling	Kim et al., 2014
COR27	AT5G42900	Nuclear protein	Yes	Yes	Photomorphogenesis, circadian clock, flowering	Kahle et al., 2020; Li et al., 2020; Zhu et al., 2020
COR28	AT4G33980	Nuclear protein	Yes	Yes	Photomorphogenesis, circadian clock, flowering	Kahle et al., 2020; Li et al., 2020
CRY2	AT1G04400	Cryptochrome	Yes	Yes	Light perception	Weidler et al., 2012; Liu et al., 2016
EBF1	AT2G25490	F-box protein	Yes	Nd	Ethylene signaling	Shi et al., 2016
EBF2	AT5G25350	F-box protein	Yes	Nd	Ethylene signaling	Shi et al., 2016
ELF3	AT2G25930	Nuclear protein	Yes	Nd	Circadian clock, flowering	Yu et al., 2008
GAI/RGA2	AT1G14920	DELLA domain protein	Yes	Yes	GA signaling	Blanco-Touriñán et al., 2020a
GATA2	AT2G45050	GATA transcription factor	Yes	Nd	Photomorphogenesis	Luo et al., 2010
GI	AT1G22770	Nuclear protein	Indirect	Nd	Circadian clock, flowering	Yu et al., 2008; Jang et al., 2015
HEC2	AT3G50330	bHLH transcription factor	Yes	Yes	Photomorphogenesis	Kathare et al., 2020
HFR1	AT1G02340	bHLH transcription factor	Yes	Nd	Photomorphogenesis, shade avoidance	Duek et al., 2004; Jang et al., 2005; Yang et al., 2005
HRT	AT5G43470	Resistance (R) protein	Yes	Nd	Plant defense	Jeong et al., 2010
HY5	AT5G11260	bZIP transcription factor	Yes	Yes	Photomorphogenesis	Osterlund et al., 2000; Saijo et al., 2003; Wang et al., 2020
HYH	AT3G17609	bZIP transcription factor	Yes	Nd	Photomorphogenesis	Holm et al., 2002
ICE1	AT3G26744	bHLH transcription factor	Yes	Nd	Stomatal differentiation, cold responses	Lee et al., 2017
ICE2/SCRM2	AT1G12860	bHLH transcription factor	Yes	Nd	Stomatal differentiation, cold responses	Lee et al., 2017
LAF1	AT4G25560	MYB transcription factor	Yes	Nd	Photomorphogenesis	Seo et al., 2003
MYC2/JAI1	AT1G32640	bHLH transcription factor	Yes	Nd	Photomorphogenesis, JA signaling	Chico et al., 2014
PAP2/MYB90	AT1G66390	MYB transcription factor	Yes	Yes	Anthocyanin biosynthesis	Maier et al., 2013; Ponnu et al., 2019
PAR1	AT2G42870	bHLH transcription factor	Yes	Nd	Photomorphogenesis	Zhou et al., 2014
PAR2	AT3G58850	bHLH transcription factor	Yes	Nd	Photomorphogenesis	Zhou et al., 2014
PCH1	AT2G16365	F-box protein	Yes	Nd	Phytochrome signaling	Cheng et al., 2020
PCHL	AT4G34550	F-box protein	Yes	Nd	Phytochrome signaling	Cheng et al., 2020
PHYA	AT1G09570	Phytochrome	Yes	Yes	Light perception	Seo et al., 2004; Saijo et al., 2008; Debrieux et al., 2013; Sheerin et al., 2015
PHYB	AT2G18790	Phytochrome	Yes	Yes	Light perception	Jang et al., 2010; Ni et al., 2014; Lu et al., 2015; Sheerin et al., 2015
PIF1	AT2G20180	bHLH transcription factor	Yes	Yes	Photomorphogenesis	Zhu et al., 2015
PIF5	AT3G59060	bHLH transcription factor	Yes	Yes	Photomorphogenesis, shade avoidance	Pham et al., 2018a
PIF8	AT4G00050	bHLH transcription factor	Yes	Nd	Photomorphogenesis	Oh et al., 2020
PIL1	AT2G46970	bHLH transcription factor	Yes	Nd	Photomorphogenesis	Luo et al., 2014
RGA/RGA1	AT2G01570	DELLA domain protein	Yes	Yes	GA signaling	Blanco-Touriñán et al., 2020a
SCAR1	AT2G34150	SCAR family protein	Yes	Nd	Root growth	Dyachok et al., 2011
SIZ1	AT5G60410	SUMO E3 ligase	Yes	Nd	Photomorphogenesis, hormonal signaling, flowering, abiotic stress responses	Kim et al., 2016
SPA2	AT4G11110	Serine/threonine kinase	Yes	Yes	Photomorphogenesis	Chen et al., 2015
SRS5	AT1G75520	RING finger-like zinc finger protein	Yes	Nd	Photomorphogenesis	Yuan et al., 2018
WDL3	AT3G23090	Microtubule regulatory protein	Yes	Nd	Photomorphogenesis, hypocotyl elongation	Lian et al., 2017

*List of proteins targeted by COP1/SPA E3 ubiquitin ligase. Fourth column is based on *in vivo*/*in vitro* data from the literature. Nd, not determined.*

**FIGURE 1:**
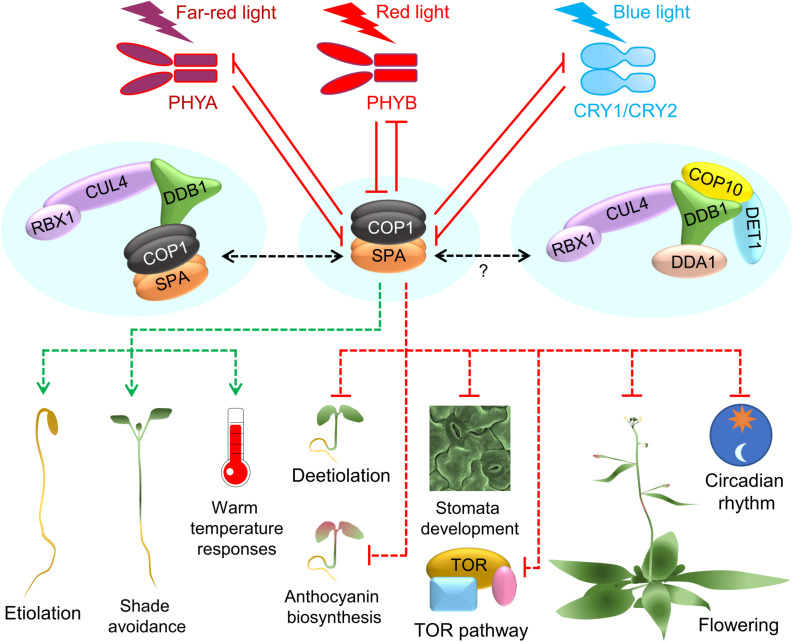
COP1/SPA acts as a central regulator of plant growth and development. A simplified schematic showing the major functions of the COP1/SPA complex as a repressor of light signaling in darkness. Phytochrome and cryptochrome photoreceptors inhibit COP1/SPA activity in the light. When photoreceptors are inactive, COP1/SPA polyubiquitinates a number of substrates and thereby promotes hypocotyl elongation, skotomorphogenesis, warm temperature and shade avoidance responses, but suppresses deetiolation, anthocyanin biosynthesis, stomata development, TOR kinase activity, circadian rhythm and flowering. The Arabidopsis COP1/SPA E3 ligase functions as part of a CUL4-DDB1-RBX1 complex and co-acts with the C3D complex. Green and red arrows represent promotion and suppression, respectively. Solid lines show direct regulation, and the dotted lines show indirect regulation. Question mark (?) shows the mechanisms that are not yet well understood.

The COP1/SPA complex acts as a central repressor of light signaling in darkness, chiefly by ubiquitinating and thereby promoting the degradation of positive regulators of photomorphogenesis, which mostly are transcription factors. When plants are exposed to light, the photoreceptors suppress COP1/SPA activity, resulting in the stabilization of the COP1/SPA substrates which subsequently promote photomorphogenesis (Hoecker, 2017; Podolec and Ulm, 2018; Ponnu, 2020). COP1/SPA proteins function as part of a CULLIN4-DAMAGED DNA BINDING PROTEIN 1 (CUL4-DDB1)-based multi-subunit, higher-order E3 ligase complex in Arabidopsis (Chen et al., 2010). Depending on the proteins that are targeted for degradation, COP1/SPA regulates various light-promoted developmental processes in plants such as hypocotyl growth, anthocyanin biosynthesis, shade avoidance, flowering time, hormone signaling, and stomata development (Duek et al., 2004; Jang et al., 2005, 2008; Yang et al., 2005; Laubinger et al., 2006; Liu et al., 2008; Lau and Deng, 2012; Maier and Hoecker, 2015; Lee et al., 2017; Wang et al., 2019; Figure 1; Table 1). Besides facilitating the degradation of proteins that promote photomorphogenesis, the COP1/SPA complex stabilizes PHYTOCHROME INTERACTING FACTORs (PIFs) in darkness, which are negative regulators of light signaling (Bauer et al., 2004; Ling et al., 2017; Pham et al., 2018b).

Generally, COP1/SPA is an inhibitor of light signaling that is active primarily in darkness. However, in UVB light, COP1 promotes photomorphogenesis by interacting with the UVB receptor UV RESISTANCE LOCUS 8 (UVR8) which leads to the stabilization of HY5 (Oravecz et al., 2006; Liang et al., 2019). Moreover, COP1/SPA facilitates the light-induced degradation of PIFs by associating with phytochromes (PHYs) (Zhu et al., 2015; Pham et al., 2018b). Furthermore, COP1/SPA targets light-activated photoreceptors for degradation, such as PHYA, PHYB and CRYPTOCHROME2 (CRY2) under far red, red and blue light, respectively (Figure 1; Seo et al., 2004; Jang et al., 2010; Weidler et al., 2012; Debrieux et al., 2013; Liu et al., 2016).

Recent years have witnessed many breakthroughs in COP1 research. In this review, we provide an update on the structure and functions of COP1 and discuss the latest developments. Importance is given to the recent works performed in Arabidopsis COP1, with a small section dedicated to mammalian COP1. This review is intended to give a bird’s-eye view of COP1 research and the readers are requested to refer to the in-depth reviews written on specific topics.

## Domains and Structure of the COP1/SPA E3 Ligase

Arabidopsis COP1 is a 76.2 kDa protein encoded by a single gene. It contains three distinct domains – the N-terminal zinc-binding RING domain, the central helical coiled-coil (CC) domain, and the C-terminal region (COP1-WD) containing tryptophan-aspartic acid repeats (WD40). The RING domain may interact with an ubiquitin conjugating enzyme E2, the CC domain confers the homodimerization of COP1 and heterodimerization with SPA proteins, and the COP1-WD binds the ubiquitination targets and photoreceptors (Deng and Quail, 1992; von Arnim and Deng, 1993; Torii et al., 1998; Uljon et al., 2016; Hoecker, 2017). The N-terminus of COP1 comprising the RING and CC domains (amino acids 1-282) is indispensable for COP1 function and can suppress the lethality of the *cop1*-*5* null mutant when overexpressed (McNellis et al., 1994; Stoop-Myer et al., 1999; Stacey et al., 2000). A number of proteins were shown to interact with the N-terminus of COP1 including COP10, an E2 variant that also is part of a CUL4-based E3 complex (Suzuki et al., 2002; Yanagawa et al., 2004). While COP10, COP1-INTERACTING PROTEIN 8 (CIP8), and MIDGET (MID) bind specifically the RING domain of COP1, other CIP proteins (CIP1, CIP4 and CIP7) and the COP1-SUPPRESSOR 2 (CSU2) associate with the COP1-CC (Matsui et al., 1995; Yamamoto et al., 1998, 2001; Torii et al., 1999; Schrader et al., 2013; Xu et al., 2015). A single bipartite nuclear localization signal (NLS) between CC and WD, and a long cytoplasmic localization signal situated in the N-terminus facilitate light-mediated nuclear import and export of COP1 (von Arnim and Deng, 1994; Stacey et al., 1999; Subramanian et al., 2004). Nuclear-localized GFP-COP1 exhibits characteristic punctate structures called speckles or nuclear bodies in which interacting proteins also colocalize (Stacey et al., 1999).

A break-through crystallization study showed that the WDs of plant and mammalian COP1 have a similar seven-bladed β-propeller configuration (Uljon et al., 2016). An intact COP1-WD is essential for COP1 function *in vivo* which is consistent with its role in substrate recognition (McNellis et al., 1994; Uljon et al., 2016). Many substrates of COP1 share a short, conserved valine-proline (VP) motif as the COP1-WD binding site (Holm et al., 2001; Laubinger et al., 2006; Uljon et al., 2016; Lau et al., 2019; Ponnu et al., 2019; Yadav et al., 2020). This mechanism was also adopted by the photoreceptors, UVR8 and CRYs, which interact with COP1 via their conserved VP motifs (Lau et al., 2019; Ponnu et al., 2019). To accommodate diverse VP motifs of a wide range of interaction partners, the COP1-WD evolved an array of conserved amino acids that form a flexible VP-binding pocket (Uljon et al., 2016; Lau et al., 2019). Interestingly, the interaction partners of mammalian COP1, such as the human TRIB1 (homolog of Drosophila tribbles) can also bind Arabidopsis COP1-WD via its VP-motifs, pointing to the coevolution of the COP1-VP-binding pocket along with the VP-containing proteins (Uljon et al., 2016). In addition to substrates and photoreceptors, COP1-WD binds DDB1 in the CUL4 complex (Chen et al., 2010).

In contrast to a single COP1 protein, Arabidopsis has four SPA proteins (SPA1 to SPA4) which have partially redundant, but also distinct functions (Hoecker et al., 1999; Laubinger et al., 2004, 2006; Menon et al., 2016). All four SPAs carry similar domains, with a CC and WD-repeat comparable to that of COP1 (Hoecker, 2017). But instead of a RING domain in COP1, SPA proteins have a weakly conserved N-terminal kinase-like domain. At least for SPA1, the kinase activity of the N-terminal domain was demonstrated recently (Paik et al., 2019; Wang et al., 2020). Accordingly, missense mutations in the kinase domain severely compromise SPA1 function in transgenic Arabidopsis plants (Holtkotte et al., 2016; Paik et al., 2019). The N-terminal domains of SPA1 and SPA2 are also responsible for the destabilization of these SPA proteins (Fittinghoff et al., 2006; Yang and Wang, 2006; Chen et al., 2016). Like the respective domains of COP1, the CC of SPA1 engages in homo- and heterodimerization (with other SPAs and with COP1), and the WD participates in substrate interactions. However, in contrast to COP1, detailed structural information is lacking for SPA-WD as no crystallization studies have been successful to date. Transgenic plants expressing individual point mutations in SPA1-WD revealed the indispensable functions of many residues in this region (Yang and Wang, 2006). In fact, the SPA1-WD contains the same conserved amino acids that make up the COP1-VP-binding pocket, suggesting that SPA proteins also engage in VP-mediated interactions (Ponnu et al., 2019). Consistent with this hypothesis, the C-termini of CRYs interact with SPA1-WD via their VP motifs (Ponnu et al., 2019).

One of the functional COP1/SPA complexes is likely a heterotetramer consisting of a COP1 homodimer with different combinations of two SPA proteins (SPA homo/heterodimers) (Zhu et al., 2008). Since photoreceptor-mediated suppression of COP1 activity involves direct interactions of light-activated photoreceptors with COP1 and SPA proteins (Podolec and Ulm, 2018), a COP1/SPA tetramer may interact with a tetramer of photoreceptors. In agreement with this idea, the Arabidopsis CRYs were recently demonstrated to form blue light-induced homo- and heterooligomers (Liu Q. et al., 2020; Shao et al., 2020; Palayam et al., 2021). A possible scenario of CRY-COP1/SPA interactions might be the binding of CRY-homo- or heterotetramers with a COP1/SPA heterotetramer, with each photoreceptor monomer in direct association with a COP1 or SPA monomer. In-depth structural and proteomic studies are required to confirm the presence of such large protein complexes *in planta*.

## COP1/SPA as a Part of a CUL4-Based E3 Ligase

There is very good evidence that in both plants and animals, COP1 acts as a part of a CUL4-based E3 ligase consisting of the core proteins CUL4, DDB1 and a RING-BOX protein (RBX) (Wertz et al., 2004; Chen et al., 2006). CUL4 is a scaffold protein anchoring a DDB1-based adaptor module and the E2-recruiting protein RBX1 at its N and C termini, respectively. In the adaptor module, DDB1 forms the core linker that attaches DWD box-containing proteins as substrate receptors. A wide range of DWD proteins that interact with DDB1 provide functional specificities to the CUL4-DDB1 E3 ligases (Hua and Vierstra, 2011). *In vitro* biochemical experiments showed that COP1/SPA proteins function as DWD proteins and associate with DDB1 via their DWD boxes to form a CUL4-COP1/SPA complex (Chen et al., 2010). However, the crystal structure of COP1-WD revealed that the DWD box is buried within and cannot be accessed without destroying the WD (Uljon et al., 2016). Hence, further studies are necessary to decipher the details of specific interactions between COP1/SPA and DDB1.

In addition to the observed DDB1-COP1/SPA protein-protein interactions, genetic evidence also strongly supports that COP1/SPA acts as part of a CUL4-based E3 ubiquitin ligase. Viable, hypomorphic *cul4* cosuppression lines exhibit constitutive photomorphogenesis in darkness and early flowering phenotypes similar to those of *cop1* and *spa* mutants. Moreover, cosuppression of *CUL4* synergistically interacts with a weak mutation in *COP1* (Chen et al., 2010). Also, COP1/SPA action in PIF degradation or thermomorphogenesis involves CUL4 (Delker et al., 2014; Zhu et al., 2015; Gangappa and Kumar, 2017). Unclear, however, is the role of the RING finger in COP1, since the COP1 RING finger, like RBX1, can bind E2 (Schulman et al., 2000; Zheng et al., 2002; Choi et al., 2014), suggesting redundant activities in the CUL4-RBX1-DDB1-COP1/SPA complex. Indeed, *in vitro*, recombinant COP1 has ubiquitin ligase activity via its RING finger domain without the need for other CUL4 complex components (Saijo et al., 2003; Seo et al., 2003). In agreement with this finding, the RING finger of COP1 is essential for COP1 function *in vivo* (Torii et al., 1998; Stoop-Myer et al., 1999). It is therefore possible that COP1/SPA proteins may not always occur in association with the CUL4-RBX1-DDB1 complex but may also act as a CUL4-independent E3 ubiquitin ligase.

Not well understood is the co-action of COP1 with DET1, another essential protein in the suppression of photomorphogenesis in darkness (Pepper et al., 1994). DET1 associates with DDB1, COP10, and DDB1-ASSOCIATED 1 (DDA1) to form a COP10-DDB1-DET1-DDA1 (C3D) complex that binds CUL4 and functions as a CUL4-based E3 ligase (Chen et al., 2006; Irigoyen et al., 2014; Fonseca and Rubio, 2019). Since Arabidopsis COP1 interacted with DDB1 independently of DET1, it was proposed that the CUL4-DDB1-COP1/SPA complex may be structurally distinct from the CUL4-C3D complex (Chen et al., 2010; Lau and Deng, 2012; Irigoyen et al., 2014). However, a recent preprint demonstrated an *in vivo* association of COP1/SPA proteins with the C3D complex in dark-grown Arabidopsis cell cultures that constitutively express DET1 (Cañibano et al., 2020). This argues for the possibility that COP1/SPA proteins may form a part of the DET1-containing C3D complex in Arabidopsis. The direct interaction between COP1 and COP10, a component of the C3D complex (Suzuki et al., 2002), may support this idea. Also, multiple pieces of evidence show that DET1 and COP1/SPA act in concert in regulating photomorphogenesis and thermomorphogenesis (Chen et al., 2010; Delker et al., 2014; Gangappa and Kumar, 2017). *det1* and *cop1* mutants have similar pleiotropic phenotypes with highly comparable gene expression patterns, such as the constitutive and abnormally high expression of light-responsive genes (Mayer et al., 1996; Ma et al., 2003). A weak allele of *det1*, acting as an enhancer of a *spa1* mutation, provided further evidence for DET1-COP1/SPA co-action throughout the developmental stages of plants (Nixdorf and Hoecker, 2010). In agreement with this, double mutant and biochemical analyses showed that CUL4 and DET1 act synergistically with COP1 in repressing photomorphogenesis (Chen et al., 2010). Further studies are required to decipher the molecular mechanism involved in the co-action between COP1/SPA proteins and the components of the C3D complex.

## Functions of COP1/SPA in Seedling Etiolation

Dark-grown seedlings undergo skotomorphogenesis and show etiolation characterized by an elongated hypocotyl, closed and yellow cotyledons, and a tightly folded apical hook. The elongated hypocotyl increases the likelihood that a soil-covered seedling reaches the light. Hence, energy is diverted from cotyledon development to elongation growth. Once exposed to light, hypocotyl elongation is inhibited, hook and cotyledons open, and chlorophyll is synthesized, i.e., the seedling deetiolates. COP1/SPA prevents deetiolation in darkness by marking several transcription factors for degradation. This includes HY5, HY5 HOMOLOG (HYH) and LONG HYPOCOTYL IN FAR-RED 1 (HFR1) and a number of other positive regulators of the light responses. In *spa* quadruple and *cop1* mutants, these transcription factors also accumulate in darkness, causing constitutive photomorphogenesis in complete darkness (Deng et al., 1991; Laubinger et al., 2004; Ordoñez-Herrera et al., 2015).

HY5 is a bZIP transcription factor that was recently shown to function mainly by activation of gene expression rather than by gene repression (Burko et al., 2020). Along with HYH and HFR1, HY5 suppresses the elongation of hypocotyls in light-grown seedlings (Lau and Deng, 2012; Hoecker, 2017; Yuan et al., 2018; Bhatnagar et al., 2020). HY5 is a non-canonical transcriptional regulator that carries a DNA-binding domain but no transcriptional activation domain. It was postulated earlier that HY5 may act in concert with other transcription factors that provide a domain for transcriptional activation. Indeed, members of a large family of B-box transcription factors (BBXs) were recently shown to interact with HY5 and to modulate HY5 activity (Datta et al., 2007, 2008; Gangappa et al., 2013; Wei et al., 2016; Lin et al., 2018; Burko et al., 2020; Bursch et al., 2020; Song Z. et al., 2020; Zhao et al., 2020). In particular, BBX20 to BBX22 were shown to be required for HY5 activity (Bursch et al., 2020). Interestingly, like HY5, many BBX proteins are also substrates of COP1 (Vaishak et al., 2019; Yadav et al., 2020). Hence, both types of transcription factors (bZIP and BBXs) that are mostly unrelated in sequence evolved to be targeted by COP1 in darkness. Many BBXs and HY5 do share COP1-WD-binding VP motifs mediating the interaction with COP1 (Gangappa and Botto, 2014; Yadav et al., 2020). BBX proteins also regulate the expression of other BBX proteins in a feedback regulatory loop involving COP1. For example, BBX4 promotes the expression of BBX11, a negative regulator of photomorphogenesis, but BBX11 interacts with COP1 to regulate the activity of BBX4 (Liu B. et al., 2020). A systematic approach is needed to identify the BBXs that interact with the COP1/SPA complex and to study the implications of these associations on light-mediated plant development.

**FIGURE 2:**
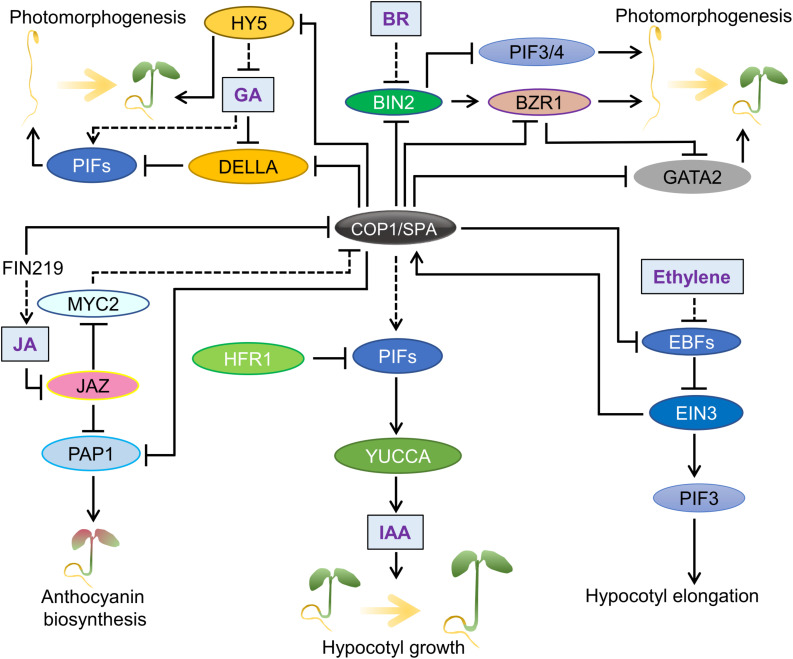
Function of the COP1/SPA complex in hormone responses. An overview of the major signaling pathways that involve hormones and the COP1/SPA complex. Effects of auxin (IAA), jasmonic acid (JA), gibberellic acid (GA), brassinosteroid (BR) and ethylene are shown. Solid lines show direct regulation and dotted lines represent indirect influence.

In addition to ubiquitinating proteins that promote photomorphogenesis, COP1/SPA stabilizes PIFs in darkness to positively regulate skotomorphogenesis. PIFs are bHLH transcription factors that, like COP1/SPA, are required for seedling etiolation in darkness (Leivar et al., 2008; Pham et al., 2018b). They upregulate many elongation-related genes (Pham et al., 2018b). Interestingly, COP1 stabilizes PIFs via a non-canonical mechanism and apparently not via its E3 ligase activity: PIF3 and PIF4 abundance is regulated by BRASSINOSTEROID-INSENSITIVE 2 (BIN2), a GSK3-like kinase. BIN2 phosphorylates PIF3 and PIF4 which leads to their degradation under dark conditions (Bernardo-García et al., 2014; Ling et al., 2017; Figure 2). The COP1/SPA complex prevents PIF phosphorylation by BIN2, thereby inhibiting the degradation of PIF3 and PIF4. While COP1 interacts with BIN2 and thereby sequesters BIN2 from binding to PIFs, SPA1 occupies the BIN2-binding domain of PIF3 (Ling et al., 2017). By stabilizing PIF3 levels, the COP1/SPA complex promotes etiolation in darkness. While suppressing photomorphogenesis by stabilizing PIFs, the COP1/SPA complex also prevents the over-accumulation of PIFs, such as PIF1, via a co-degradation mechanism involving HFR1 in darkness (Xu et al., 2017).

Under light conditions, photoreceptors such as CRYs, PHYs and UVR8 suppress the activity of the COP1/SPA complex to promote photomorphogenesis (Hoecker, 2017; Podolec and Ulm, 2018; Ponnu, 2020). As revealed by recent studies, the COP1/SPA complex also functions as a promoter of photomorphogenesis, via destabilizing PIFs under light conditions. The COP1/SPA complex mediates the light-induced degradation of PIF1 by functioning as a CUL4-COP1/SPA E3 ligase in a PHYB-dependent manner (Zhu et al., 2015). In this complex, SPA1 functions as a serine/threonine kinase and phosphorylates PIF1, which is necessary for light-induced PIF1 degradation (Paik et al., 2019). The PHOTOPERIODIC CONTROL OF HYPOCOTYL 1 (PCH1) and PCH1-LIKE (PCHL) that regulate the thermal reversion of PHYB, enhance the degradation of PIF1 in light, by promoting PHYB-PIF1 and COP1/SPA-PIF1 associations (Cheng et al., 2020).

## Functions of COP1/SPA in Shade

Plants growing under dense canopies (low red to far-red ratio) undergo adaptive changes in response to shade. These responses include enhanced hypocotyl and internode elongation, hyponasty, reduced branching and accelerated flowering, and are collectively known as shade-avoidance syndrome (SAS) (Fiorucci and Fankhauser, 2017). Both COP1 and SPA proteins are essential for most facets of the SAS, except for earlier flowering (McNellis et al., 1994; Rolauffs et al., 2012). Shade enhances elongation growth by promoting the nuclear accumulation of COP1 (Pacín et al., 2013) which subsequently promotes the degradation of HFR1, a negative regulator of shade avoidance responses. Low levels of HFR1 lead to an activation of PIF-mediated gene expression which enhances hypocotyl elongation via auxin (Pacín et al., 2016; Iglesias et al., 2018). In addition, COP1 may act via HY5 and HYH which suppress stem growth in Arabidopsis (Nozue et al., 2015). Genetic interaction between COP1 and BBX transcription factors (BBX21, BBX22 and BBX25) demonstrated the involvement of BBXs in SAS responses (Crocco et al., 2010; Gangappa et al., 2013). Hence, BBX and HY5 may act in concert to regulate the SAS. The SAS responses are strongly inhibited by UVB via UVR8-COP1 interactions, resulting in stabilization of HY5 and suppression of PIFs (Favory et al., 2009; Hayes et al., 2017).

## COP1/SPA Proteins in Temperature Responses

During their life cycle, plants are challenged with fluctuations in temperature. Extreme temperatures (frost, excessive heat) lead to stress responses, while smaller alterations lead to adaptive growth. Such adaptive growth responses to high ambient temperature (ca. 27–29°C for Arabidopsis) mimic growth responses in canopy shade: hypocotyls and internodes elongate and leaves show upward (hyponastic) growth, perhaps to cool the plant surface and to protect the meristem from soil heat (Crawford et al., 2012). Light and temperature signaling are strongly interconnected (Quint et al., 2016). Indeed, PHYB is a sensor of high ambient temperature because high temperature promotes dark reversion of PHYB from the active to the inactive conformation (Jung et al., 2016; Legris et al., 2016). Since active PHYB inhibits COP1/SPA activity, one can expect that COP1/SPA is also involved in temperature signaling. Experimental results confirm this idea: both COP1 and SPA are required for the response to high ambient temperature (Delker et al., 2014; Gangappa and Kumar, 2017; Lee et al., 2020).

At the cellular level, PIF4 coordinates light and warm temperature signals to promote hypocotyl elongation (Martínez et al., 2018; Vu et al., 2019). A DET1-COP1-HY5 hub controls PIF4 levels (Delker et al., 2014). While DET1 and COP1 stabilize PIF4 and promote hypocotyl elongation at elevated temperatures, HY5 negatively regulates thermosensory growth by competing with PIF4 targets (Gangappa and Kumar, 2017). Warm temperature-stabilized COP1/SPA complex reduces HY5 levels and stabilizes PIF4 (Legris et al., 2017; Martínez et al., 2018). Consistent with this observation, elevated temperatures were shown to promote nuclear accumulation of COP1 which leads to the suppression of HY5 activity and hypocotyl elongation (Park et al., 2017). In addition, elevated temperatures reduce anthocyanin biosynthesis via the COP1-HY5 module. Plants grown at warm temperatures accumulate less HY5 and also show reduced accumulation of anthocyanins (Kim et al., 2017). By directly binding and repressing MYB-LIK2 (MYBL2) transcription factor that negatively regulates anthocyanin biosynthesis (Matsui et al., 2008), HY5 acts as a positive regulator of anthocyanin accumulation (Wang et al., 2016). Lower HY5 at elevated temperatures leads to the enhanced expression of MYBL2, causing reduced anthocyanin biosynthesis (Kim et al., 2017). The enhancement of COP1 activity under elevated temperature seems to be tissue-specific, as COP1 was shown to be depleted at higher temperatures in the Arabidopsis rosettes which stabilizes GIGANTEA (GI) and accelerates flowering (Jang et al., 2015). In contrast, cold temperature depletes COP1 from the nuclei of root cells and causes HY5 accumulation which leads to the expression of cold-responsive genes (Catalá et al., 2011). Taken together, these observations point to the complexity of COP1-regulation by temperature signals. Future research should aim at elucidating the mechanisms that govern temperature-dependent nuclear import and export of COP1 in different tissues and at different developmental stages of plant growth.

In addition to COP1, SPA proteins are also required for PIF4-mediated thermomorphogenesis (Delker et al., 2014; Lee et al., 2020). SPAs were required for PIF4 activity, and SPA1-mediated phosphorylation of PIF4 was recently demonstrated *in vitro*. In contrast to BIN2-mediated phosphorylation that destabilizes PIF4 (Ling et al., 2017), SPA1-mediated phosphorylation appears to stabilize PIF4. Also, SPAs promote PHYB degradation in the SPA1-PHYB-PIF4 complex under warm temperatures, which may further stabilize PIF4 (Lee et al., 2020). The mechanistic details underlying the differential fates of PIF4 stability based on the phosphorylating kinases will be of interest in future research.

## The COP1/SPA Complex in Hormonal Signaling

COP1/SPA activity intersects with multiple hormone signaling pathways in the control of growth, development, and stress responses (Wang et al., 2019). In particular, responses to shade and elevated ambient temperature involve the hormone auxin. Auxin is a universal promoter of hypocotyl and petiole growth. PIF transcription factors have been shown to enhance auxin levels in the shade by directly activating auxin biosynthesis genes such as YUCCAs, and COP1/SPA is required for this activation (Rolauffs et al., 2012; Pacín et al., 2016; Iglesias et al., 2018; Figure 2). Under warm temperatures and shade conditions, COP1 stabilizes PIFs leading to an enhancement in auxin levels (Gangappa and Kumar, 2017; Park et al., 2017). In contrast, under UVB light, UVR8 interacts with COP1 to suppress the E3 ubiquitin ligase activity, leading to destabilization of PIF4 and inhibition of hypocotyl growth via reduced auxin biosynthesis (Hayes et al., 2017; Tavridou et al., 2020). COP1 regulates polar auxin transport and hence root growth, perhaps by regulating PIN-FORMED (PIN) auxin transporters (Sassi et al., 2012).

Brassinosteroids (BRs) are plant hormones that are repressors of light-mediated de-etiolation and chlorophyll biosynthesis (Gupta and Nath, 2020). Consequently, BR-insensitive and deficient mutants show constitutive photomorphogenesis in darkness (Li et al., 1996; Nam and Li, 2002; Song et al., 2009). The signaling cross-talk mediated by COP1 and the BR pathway is an emerging field (Figure 2). One of the converging points of light and BR signaling may involve the GATA2 transcription factor that positively regulates light signaling. Both COP1 and BR signaling suppress the activities of GATA2 in darkness to promote skotomorphogenesis. While COP1 targets GATA2 for degradation, BRASSINAZOLE-RESISTANT 1 (BZR1) transcription factor inhibits *GATA2* expression in darkness (Luo et al., 2010). It is possible that COP1 may affect BZR1 via the inhibition of BIN2 (Ling et al., 2017), a kinase that phosphorylates both BZR1 (He et al., 2002, 2) and PIF4 (Bernardo-García et al., 2014), and also act as a negative regulator of BR signaling. Consistent with this, elevated temperatures increase the nuclear accumulation of COP1, which sequesters BIN2 and enhances the function of PIF1 and BZR1 (Nieto et al., 2020). Alternatively, COP1 may reduce the BR sensitivity at cellular level by regulating MEMBRANE STEROID BINDING PROTEIN 1 (MSBP1) involved in suppressing the BR perception (Song et al., 2009). In darkness the activity of MSBP1 is suppressed in a COP1-dependent manner, which may increase BR perception and promote etiolation (Shi et al., 2011).

Gibberellins (GA) act similar to BR and suppress photomorphogenesis in darkness (Alabadí et al., 2004, 2008; Figure 2). GA and COP1 intersect at the DELLA proteins which are key negative regulators of GA signaling. In response to shade and elevated ambient temperatures, COP1 directly targets DELLAs for degradation and thereby promotes plant growth under these conditions (Blanco-Touriñán et al., 2020a). DELLAs are also degraded in response to GA via the SLEEPY1 (SLY1) ubiquitin ligase (Dill et al., 2004; Blanco-Touriñán et al., 2020b). SLY1 and COP1 interact with different domains in the DELLA proteins, suggesting independent mechanisms (Blanco-Touriñán et al., 2020a). DELLAs are negative regulators of PIFs, and by causing DELLA degradation, GA, promotes skotomorphogenesis by enhancing PIF activity. The COP1-HY5 pathway plays a crucial role in mediating GA-dependent skotomorphogenesis probably also via PIFs (Mazzella et al., 2014). Light promotes photomorphogenesis by suppressing GA signaling, mainly by reducing the GA content, and presumably also via regulating COP1-HY5-PIF module (Alabadí et al., 2008; Mazzella et al., 2014).

Ethylene is a gaseous hormone that acts contrastingly in dark and light. Under light conditions, ethylene promotes hypocotyl growth, but suppresses it in darkness (Yu and Huang, 2017; Figure 2). However, most of the ethylene-mediated processes in plants require light. COP1 co-operates with ethylene via stabilizing ETHYLENE INSENSITIVE 3 (EIN3), which promotes ethylene responses (Zhong et al., 2014). COP1 stabilizes EIN3 by degrading EIN3-BINDING F-BOX PROTEINs (EBFs) that target EIN3 for degradation (Shi et al., 2016). Interestingly, ethylene via EIN3 positively regulates the nuclear accumulation of COP1 in the light and thereby causes HY5 degradation to modulate hypocotyl growth (Yu et al., 2013). Taken together, the present knowledge suggests that under light conditions the COP1-HY5 module acts together with the ethylene-EIN3-PIF pathway to promote hypocotyl growth. The mechanisms of ethylene-COP1 coaction in darkness are not fully understood.

Jasmonic acid (JA), a plant hormone crucial in biotic stresses such as herbivory and pathogen attack, is also involved in the regulation of skotomorphogenesis (Huang et al., 2017; Figure 2). At lower levels of JA, JASMONATE-ZIM domain proteins (JAZs) suppress MYC transcription factors. JA degrades JAZ via recruiting an E3 ligase, leading to the activation of MYC2. In presence of light, MYC2 activates HY5. In addition, MYC2 can affect the function of both COP1 and SPA proteins (Gangappa et al., 2010; Zheng et al., 2017). Alternatively, JA can suppress the formation of the COP1/SPA complex and can attenuate the function of nuclear COP1 (Zheng et al., 2017). Whether JA-mediated COP1 suppression involves MYC2 is currently unclear. Another mode in which JA signaling affects COP1 activity and hypocotyl growth is through FAR-RED INSENSITIVE 219/JASMONATE RESISTANT 1 (FIN219/JAR1) that catalyzes the synthesis of the bioactive form of JA. FIN219 regulates hypocotyl elongation in shade conditions by promoting the cytoplasmic accumulation of COP1 (Swain et al., 2017). This action may antagonize shade-induced nuclear accumulation of COP1 (Pacín et al., 2013). Additionally, in blue light, FIN219-mediated export of COP1 from the nucleus act in concert with CRY-mediated suppression of COP1/SPA complex (Chen H.-J. et al., 2018). However, JA enhances the CRY-FIN219 association in blue light, thereby attenuating the CRY-mediated suppression of the COP1/SPA complex. This leads to enhanced COP1 activity and reduction of HY5 levels in the nucleus that promotes hypocotyl elongation (Chen H.-J. et al., 2018). JA-COP1 action is also implicated in the regulation of photomorphogenesis such as anthocyanin biosynthesis and protochlorophyllide formation (Wang et al., 2019). Similar to JA, cytokinins (CKs) promote photomorphogenesis and might act via other hormonal pathways. CKs may regulate plant growth via the COP1-HY5 module, but the underlying mechanisms remain unexplored (Vandenbussche et al., 2007).

Abscisic acid (ABA) functions opposite to light signals, antagonizing seed germination, seedling establishment, stomatal opening and root growth. ABA-INSENSITIVE 5 (ABI5), a bZIP transcription factor that plays a major role in ABA responses, colocalizes with COP1 (Lopez-Molina et al., 2003). Although direct interaction between COP1 and ABI5 is yet to be proven, genetic analyses suggest that COP1 acts downstream of ABI5 and promotes ABA signaling. COP1 positively regulates the ABA-mediated post-germination growth arrest of seedlings, by facilitating the binding of ABI5 to the promoters of ABA-responsive genes (Yadukrishnan and Datta, 2020). In addition, COP1 colocalizes with ABI5-binding protein AFP1, which promotes ABI5 degradation (Lopez-Molina et al., 2003). COP1-HY5 module also converges on ABA signaling via ABI4, another transcription factor that mediates ABA signaling. ABI4 acts antagonistically with HY5 in regulating COP1 and both ABI4 and HY5 are targeted by COP1 for degradation under dark and light conditions. Since the nuclear ABI4 is activated by a chloroplast-derived signal, COP1 integrates retrograde and light signaling pathways (Xu et al., 2016c). Besides acting at ABA-mediated seed germination and seedling establishment, COP1 participates in ABA-induced stomatal closure and functions as an important regulator of abiotic stress responses in Arabidopsis and pea (Moazzam-Jazi et al., 2018). Recently a mechanism of COP1-mediated stomatal closure via the regulation of ABA co-receptors has been described. COP1 directly interacts and facilitates the degradation of type 2C phosphatases (PP2Cs), which are ABA co-receptors that positively regulate stomatal opening in the absence of ABA. By degrading PP2Cs, COP1 promotes stomatal closure and acts together with ABA (Chen et al., 2020). The COP1-interacting protein CIP1 has also been reported to promote ABA signaling, but further details remain unknown (Ren et al., 2016).

The mechanisms underlying the activity of strigolactone (SL) in regulating plant growth and development are poorly understood. SL has been reported to stabilize HY5 via suppressing COP1 activity to promote photomorphogenesis. This is thought to occur via nuclear export of COP1 or COP1 degradation. Thus, SL may act in parallel with photoreceptors and repress COP1 (Tsuchiya et al., 2010; Jia et al., 2014). Also COP1 and HY5 associate with BBX20, a negative regulator of the SL pathway (Wei et al., 2016). The role of COP1/SPA complex in the SL pathway needs to be explored further in detail.

## Other Functions of the COP1/SPA Complex

The numerous functions of the COP1/SPA complex are mediated by an increasing number of substrates that are being identified, with each substrate having specific functions in plant growth, development, and metabolism (see also Table 1). However, in contrast to mammalian COP1, the functions of plant COP1 in cell division, cell elongation, cytoskeletal network, etc., are relatively less explored. An earlier study suggests that in darkness, COP1 inhibits root elongation in Arabidopsis seedlings by targeting and degrading SCAR, a component of cytoskeletal dynamics (Dyachok et al., 2011). A direct link between COP1 and the cortical microtubule was found also in hypocotyl cells. In darkness, COP1 binds and promotes the degradation of WAVE-DAMPENED 2-LIKE 3 (WDL3), a microtubule-associated protein that promotes cell elongation and hypocotyl elongation (Lian et al., 2017). Surprisingly, WDL3 is a cytosolic protein (Lian et al., 2017) and therefore an unusual COP1 target since COP1 activity usually depends on its nuclear localization (von Arnim et al., 1997). Another example supporting a cytoplasmic activity of the COP1/SPA complex was reported in the microRNA (miRNA) biogenesis pathway of Arabidopsis (Cho et al., 2014). *cop1* mutants are defective in miRNA biogenesis due to low levels of HYPONASTIC LEAVES 1 (HYL1), an RNA-binding protein involved in miRNA processing. HYL1 is cleaved by an unknown protein in the cytoplasm. Upon light exposure, COP1 translocates into the cytoplasm and may facilitate the degradation of this unknown protein. In this way, COP1 stabilizes HYL1 levels and thereby promotes microRNA biogenesis (Cho et al., 2014). However, the implication of possible COP1 activity in the cytosol requires further research. Interaction between COP1 and MIDGET (MID), a component of topoisomerase VI, via the very N-terminal domain of COP1 suggests that COP1 may have a direct effect on endoreduplication and genome integrity, the primary functions of MID (Schrader et al., 2013). As COP1 does not affect MID protein levels, the impact of the COP1-MID interaction in DNA replication and genome integrity is so far unclear (Schrader et al., 2013).

Mutations in *COP1*/*SPA* genes cause pleiotropic phenotypes throughout plant development (McNellis et al., 1994; Laubinger et al., 2004). COP1/SPA aligns endogenous developmental pathways with the ambient light environment by controlling the protein stability of key developmental regulators. A pivotal function of COP1/SPA in the regulation of flowering time by photoperiod involves the transcription factor CONSTANS (CO). In darkness, CO is degraded via COP1/SPA which causes a delay in flowering under non-inductive short-day conditions, and therefore allows adjustment of flowering time to seasons (Laubinger et al., 2006; Jang et al., 2008; Liu et al., 2008; Sarid-Krebs et al., 2015; Xu et al., 2016b). Consistent with this finding, Arabidopsis *cop1* mutants constitutively express floral integrator genes and undergo flowering in complete darkness when grown on sucrose-containing media (Nakagawa and Komeda, 2004). COP1 also suppresses flowering time by promoting the degradation of GI, a circadian clock-associated protein, in an EARLY FLOWERING 3 (ELF3)-dependent manner, and functions as an integrator of photoperiod and circadian signals (Yu et al., 2008). In darkness, COP1 represses TARGET OF RAPAMYCIN (TOR) kinase, the central regulator of energy signaling required for light-induced growth activation. By suppressing TOR in darkness, COP1 prevents light-induced stem cell activation at the shoot apex (Pfeiffer et al., 2016) and also the light-mediated enhancement in translation (Chen G.-H. et al., 2018).

A recent work demonstrated another mode of signal integration between light and the circadian clock via the regulation of COLD REGULATED 27 (COR27) and its homolog COR28 by the COP1/SPA complex. Both CORs are key regulators of the circadian clock and cold responses (Wang et al., 2017), and also function as negative regulators of light signaling. In darkness, CORs interact with the COP1/SPA complex via their VP motifs and thereby are degraded via the 26S proteasome (Kahle et al., 2020; Zhu et al., 2020). In light, CORs interact with HY5 and interfere with HY5 activity on the downstream genes to fine-tune hypocotyl growth in Arabidopsis (Kahle et al., 2020; Li et al., 2020; Zhu et al., 2020).

The differentiation of stomata from protodermal cells is also under the control of light and COP1/SPA. In darkness, stomata differentiation is suppressed by COP1/SPA-mediated degradation of INDUCER OF CAB EXPRESSION 1 (ICE1), a transcription factor required for stomata differentiation (Lee et al., 2017). Accordingly, *cop1* mutants accumulate higher levels of ICE1 proteins in the nuclei of abaxial leaf epidermal cells, suggesting that the interaction partners of the COP1/SPA complex may vary according to the tissue types. In addition to stomatal differentiation, COP1 promotes ABA-mediated stomatal closure (Chen et al., 2020) and functions as an important regulator of abiotic stress in plants (Moazzam-Jazi et al., 2018). The role of COP1 in biotic stresses has also been documented. COP1 negatively regulates defense against Turnip Crinkle Mosaic Virus (TMV) in darkness by interacting and marking the resistance protein HYPERSENSITIVE RESPONSE TO TCV (HRT) for degradation (Jeong et al., 2010). Paradoxically, COP1 also contributes to the HRT-mediated defense against TMV by promoting the stability of RNA-binding proteins that are required for HRT-mediated TMV resistance (Lim et al., 2018). COP1/SPA also regulates specific metabolic pathways in darkness, such as anthocyanin biosynthesis, by targeting the PRODUCTION OF ANTHOCYANIN PIGMENT (PAP) transcription factors for degradation (Maier et al., 2013).

## Regulation of COP1/SPA Activity

Upon illumination, plant photoreceptors suppress the E3 ligase activity of the COP1/SPA complex by directly interacting with COP1 and SPA proteins. This leads to a stabilization of COP1/SPA substrates and subsequent photomorphogenesis. Diverse mechanisms underlying the suppression of COP1/SPA by photoreceptors have been studied in detail, such as the nuclear export of COP1, dissociation of the COP1-SPA interaction, light-mediated degradation of SPA proteins, prevention of COP1 dimerization, and VP-mediated displacement of substrates by photoreceptors. Readers are encouraged to refer to recent reviews discussing these mechanisms in detail (Menon et al., 2016; Hoecker, 2017; Podolec and Ulm, 2018; Ponnu, 2020).

Genetic evidence indicates that both COP1 and SPA proteins are required for COP1/SPA activity in the suppression of photomorphogenesis in darkness. *In vitro*, COP1 alone has catalytic activity as a ubiquitin ligase due to the presence of an E2-binding zinc finger, implying that SPA proteins may have an essential regulatory function on the activity of COP1. SPA proteins are not necessary for nuclear accumulation of COP1 in darkness, as COP1 accumulates normally in the nucleus in dark-gown *spa* null mutants (Balcerowicz et al., 2017). On the other hand, SPA proteins via their DDB1-binding WD repeats contribute to the formation of a CUL4-DDB1-RBX1 ubiquitin ligase. *In vitro* experiments demonstrating catalytic activity of CUL4-DDB1-RBX1-COP1/SPA complexes in the presence and absence of SPAs are necessary to investigate the role of SPA proteins for such an E3 ligase. Since PIFs and COP1/SPA are both required to suppress photomorphogenesis in darkness, they might act together. Indeed, PIF1 was shown to interact with the WD-repeat of COP1 and SPA1. Moreover, *pif* and *cop1/spa* mutations have synergistic effects in dark-grown seedlings and the COP1/SPA target HY5 is not degraded in dark-grown *pif* mutants. Also, PIF1 enhances COP1 ubiquitination activity *in vitro* (Xu X. et al., 2014). The mechanistic nature of possible PIF-COP1/SPA co-action is so far unknown.

Since SPA proteins are specific to the green lineage, they may have evolved to place the activity of COP1 under the control of light, i.e., to allow inhibition of COP1 activity by light. Multiple pieces of evidence support this idea. Though both COP1 and SPA proteins can interact with photoreceptors, SPA proteins may regulate the affinity *in vivo*. Indeed, SPA proteins are required for the interaction of CRY1 with COP1 *in vivo* (Holtkotte et al., 2017). SPA proteins are also required for the light-induced nuclear exclusion of COP1 and thereby contribute to the light-induced inhibition of COP1 (Balcerowicz et al., 2017). Moreover, red and blue light leads to a dissociation of the COP1-SPA1 interaction, likely a very important mechanism of light-induced inhibition of COP1/SPA activity (Lian et al., 2011; Liu et al., 2011; Lu et al., 2015; Sheerin et al., 2015). Light leads to destabilization of SPA1 and very rapid degradation of SPA2 which will limit COP1 activity in the light (Balcerowicz et al., 2011; Chen et al., 2015, 2016). The kinase activity of SPA1 promotes photomorphogenesis by red light-induced phosphorylation, ubiquitination and subsequent degradation of PIF1 in a PHYB-dependent fashion (Paik et al., 2019).

Though COP1 protein levels do not change even after prolonged exposure to light (Zhu et al., 2008; Balcerowicz et al., 2011), COP1 homeostasis is controlled in darkness by CSU1, a RING-E3 ligase that was identified in a mutant screen for *cop1* suppressors. CSU1 interacts with COP1, polyubiquitinates it, and thereby reduces COP1 levels in darkness. Similarly, CSU1 down-regulates SPA1 levels in darkness as well (Xu D. et al., 2014). CSU2, identified in the same suppressor screen, inhibits COP1 activity via a different mechanism. The interaction between CSU2 and COP1 via their respective CC domains suppresses COP1 E3 ligase activity *in vitro*. It is speculated that CSU2 may interfere with COP1 homodimerization or COP1/SPA heterodimerization (Xu et al., 2015). A mutation in *CSU4* on the other hand genetically suppresses the *cop1* mutation by interacting with CIRCADIAN CLOCK-ASSOCIATED 1 (CCA1) (Zhao et al., 2018). Another mechanism of COP1 regulation may be via the alternatively spliced COP1b, which potentially interferes with COP1 activity, at least in certain tissues (Zhou et al., 1998). Recently, the C3D component DET1 was also shown to regulate the levels of COP1 toward fine-tuning HY5 activity in Arabidopsis (Cañibano et al., 2020). However, the mechanisms underlying DET1-mediated destabilization of COP1 are not yet known.

Post-translational modifications also regulate COP1 activity. The attachment of small ubiquitin-like modifier (SUMO) peptides via the direct interaction of the SUMO E3 ligase SAP AND MIZ1 DOMAIN- CONTAINING LIGASE1 (SIZ1), enhances the COP1 E3 ligase activity toward HY5, but also leads to SIZ1 ubiquitination and proteasome-mediated degradation (Lin et al., 2016). *csu3* suppressor of *cop1* carried mutations in the serine/threonine kinase PINOID (PID), which regulates auxin homeostasis by phosphorylating auxin efflux carriers (Berkel et al., 2013). PID directly interacts and phosphorylates COP1 at serine 20, which suppresses COP1 activity (Lin et al., 2017).

Nuclear import and export of COP1 play an important role in regulating COP1 activity (von Arnim and Deng, 1994). Light, temperature, shade, and hormonal responses influence COP1 localization and thereby affect COP1 activity. While exposure to light promotes nuclear export of COP1 (Pacín et al., 2014, 1; Podolec and Ulm, 2018), warm temperature and shade promote nuclear accumulation of COP1 (Pacín et al., 2013; Park et al., 2017). Plant hormonal pathways involving auxin and JA regulate COP1 activity mainly via FIN219. Auxin induces *FIN219* expression, which catalyzes JA biosynthesis (Hsieh and Okamoto, 2014). Under shade conditions, FIN219 directly interacts with COP1 and promotes its nuclear export (Swain et al., 2017).

## Cop1 in Animals: A Comparison With Arabidopsis Cop1

**FIGURE 3:**
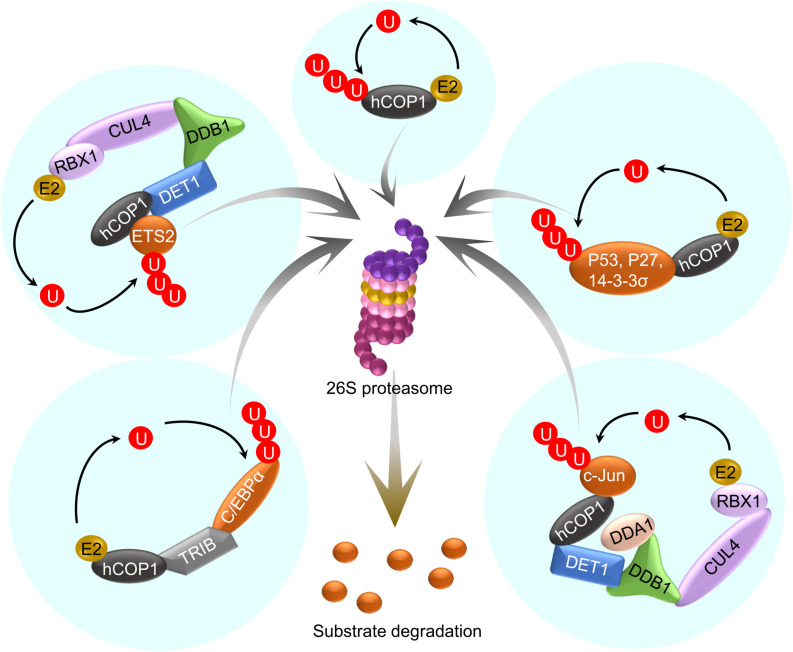
hCOP1 acts as part of multiple E3 ubiquitin ligases. A simplified representation of the hCOP1 function. hCOP1 itself acts as an E3 ligase and also cooperates with other E3 ligases in ubiquitinating and thereby promoting the degradation of different substrates.

Mammalian COP1, especially human COP1 (hCOP1) was identified as an ortholog of Arabidopsis COP1 (referred to as AtCOP1 in this section) that binds to the bZIP transcription factor c-Jun, an oncogenic protein (Bianchi et al., 2003). hCOP1 contains very similar domains as AtCOP1, i.e., a RING finger domain, a coiled-coil domain and a WD repeat. Like AtCOP1, hCOP1 is encoded by a single gene and functions as an E3 ubiquitin ligase, but in contrast to AtCOP1, it recognizes different targets which regulate cell division, DNA repair and apoptosis (Dornan et al., 2004; Song Y. et al., 2020). Various studies show that hCOP1 may be a potential oncogenic factor or a tumor suppressor, owing to its diverse roles in tumorigenesis (Lee et al., 2010). Several substrates and interacting proteins of hCOP1 were identified over the years (Song Y. et al., 2020). Hence, like AtCOP1, hCOP1 targets multiple substrates, and thereby, regulates multiple processes (Figure 3).

Many functional parallels and differences can be drawn between AtCOP1 and hCOP1. Similar to AtCOP1, hCOP1 is nuclear-localized. Upon DNA damage, hCOP1 undergoes phosphorylation and autoubiquitination followed by degradation (Dornan et al., 2006). The phosphorylated residues of hCOP1 are not conserved in AtCOP1, suggesting that this mechanism of regulation is not conserved in AtCOP1. *In vitro* phosphorylation of AtCOP1 at Ser20, mediated by PID kinase, is so far the only evidence for COP1 phosphorylation (Lin et al., 2017). Also, though observed *in vitro* (e.g., Seo et al., 2003), there is no functional evidence for a biological role of AtCOP1 autoubiquitination. However, COP1-dependent degradation of SPA2 in light-grown seedlings was reported (Chen et al., 2015).

Like AtCOP1, hCOP1 is part of higher-order CUL4-RBX1-DDB1-based E3 ligase complex. However, unlike in Arabidopsis, the association between hCOP1 and DDB1 in the CUL4-E3 complex is not direct, but bridged by DET1 (Wertz et al., 2004). Work in a recent preprint demonstrated that AtCOP1 co-purifies with AtDET1 *in vivo*, raising the possibility that AtCOP1 acts together with AtDET1 as members of the same complex or of interacting complexes (Cañibano et al., 2020). Depending on the ubiquitination substrates, hCOP1 could act as an E3 ligase independently or in association with the CUL4-DDB1-RBX-DET1 (CDD) complex. hCOP1 ubiquitinates and promotes the degradation of the tumor suppressor protein p53 by interacting via the RING-domain, without involving any other E3 ligases (Dornan et al., 2004). By mediating the degradation of p53, hCOP1 acts as a suppressor of tumorigenesis. Similarly, hCOP1 interacts with another tumor suppressor protein p27 via the RING-domain that results in polyubiquitination and degradation of p27 (Choi et al., 2015). Unlike with p53 and p27, hCOP1 could directly or indirectly associate with CDD complex to degrade targets such as c-Jun. Even though the interaction between hCOP1 and c-Jun occurs via hCOP1-WD, the N-terminus of hCOP1 is essential for ubiquitination and degradation of c-Jun (Bianchi et al., 2003). Whether hCOP1 ubiquitinates c-Jun independently of CDD remains to be investigated. As the N-terminus is indispensable also for AtCOP1 function (Torii et al., 1998; Stoop-Myer et al., 1999), further research is required to determine whether AtCOP1 could act as an E3 ligase independently of the CUL4-DDB1 complex. Similarly, PEA3, an ETS family protein regulating cell proliferation, interacts with the CC and WD of hCOP1 and is subjected to ubiquitination and degradation via the CDD complex (Baert et al., 2010). ETS2, another member of the ETS family, is also targeted by hCOP1 to degradation, mediated by the CDD complex. However, similar to the interaction with c-Jun, an intact RING domain is essential for ETS2 degradation, raising the possibility that a part of ETS2 ubiquitination may occur via hCOP1 alone (Carrero et al., 2016). In addition, unlike c-Jun, DET1 of the CDD complex directly interacts with ETS2. hCOP1 also suppresses ETS1, but the involvement of the CDD complex is not reported so far. hCOP1 targets many other proteins for degradation such as MTA and FOXO; however, the mechanistic details are still unclear. Taken together, the N-terminus of both AtCOP1 and hCOP1 are essential for their functions.

hCOP1 also targets and promotes the degradation of C/EBPα, another tumor suppressor protein, via Tribbles (TRIB) as adapter proteins. Similarly, the interaction between AtCOP1 and GI is mediated by ELF3 as a bridge protein (Yu et al., 2008). Interestingly, ELF3 is also targeted for degradation by COP1 via another bridge protein BBX19 (Wang et al., 2015). hCOP1-WD and AtCOP1-WD both co-crystallized with TRIB peptides, indicating that the VP-binding pockets of AtCOP1 and hCOP1 are very similar and both are capable of associating with diverse VP domain-containing proteins (Uljon et al., 2016; Lau et al., 2019). TRIBs are pseudo serine/threonine protein kinases that do not usually phosphorylate the substrates on their own but instead function as scaffold proteins involved in protein binding (Eyers et al., 2017). TRIB1 also regulates hCOP1 activity via promoting nuclear accumulation of COP1. Interestingly, an intramolecular interaction within hCOP1 involving a pseudo-substrate latch and the VP-binding pocket of hCOP1 promotes the nuclear export of hCOP1. TRIB1 via its VP motif interacts with the VP-binding pocket of hCOP1, displacing the pseudo-substrate latch from hCOP1-WD, thereby masking the nuclear export signal (Kung and Jura, 2019). Future research could explore similar mechanisms that may operate in AtCOP1. CRYs are important regulators of AtCOP1 under blue light conditions (Holtkotte et al., 2017; Ponnu et al., 2019; Ponnu, 2020). However, hCRYs that function in circadian regulation do not interact directly with hCOP1. Recently, hCRYs are shown to disrupt the association between hCOP1 and C3D complex by directly interacting with DET1, suggesting the evolutionary conservation of CRY-mediated repression of COP1 E3 ligase activity (Rizzini et al., 2019).

## Conclusion and Perspectives

Almost every facet of plant growth and development directly or indirectly associates with photomorphogenesis. The tremendous progress made in the last decades to understand the plant responses to light signals has unraveled many previously unknown mechanisms and established new signaling interconnections. Many important pathways that govern plant survival and establishment share the functions of key players that converge on the COP1/SPA complex. An increasing number of substrates and interaction partners that are being discovered connects COP1/SPA to almost every aspect of a plant’s lifecycle. Genetic, molecular, biochemical, and structural breakthroughs in recent times have provided new insights into the functions of the COP1/SPA E3 ligase.

However, many exciting and crucial questions remain to be answered, such as whether the COP1/SPA complex can function as an E3 ligase alone or only as a part of CUL4-based E3 complexes. Moreover, while both COP1 and SPA proteins are part of the CUL4-DDB1-RBX1 complex, they may also associate with the C3D complex. Notably, the mutants in the components of CUL4-based E3 complexes, including DET1, exhibit constitutive photomorphogenesis in darkness. As hCOP1 acts together with hDET1, it remains to be explored how AtDET1 and AtCOP1/AtSPA co-act in plants. Given that the COP1/SPA complex likely functions as a heterotetramer, large multimeric complexes may be formed *in vivo* when associated with CUL4-based E3 ligases. Therefore, it is likely that photoreceptors suppress the activity of these large complexes via additional mechanisms that are not known so far. In addition, such multimeric complexes may have functional specificities in tissue and developmental contexts. The pathways that affect these specificities and their implications are worth exploring.

The recent discovery that SPA1 functions as a serine/threonine kinase will further advance our understanding of the role of SPA proteins in the COP1/SPA complex. Future research may explore the kinase activity of SPAs other than SPA1, processes that regulate SPA1 kinase activity and additional phosphorylation substrates of the SPA1 kinase. Since hCOP1 shares similarities with AtCOP1, interdisciplinary comparative studies employing advanced biochemical and structural tools may provide more insights into the functions of the COP1/SPA E3 ubiquitin ligase.

## Author Contributions

Both authors listed have made a substantial, direct, and intellectual contribution to the work and approved it for publication.

## Conflict of Interest

The authors declare that the research was conducted in the absence of any commercial or financial relationships that could be construed as a potential conflict of interest.

## References

[B1] Alabadí D. Gallego-Bartolomé J. Orlando L. García-Cárcel L. Rubio V. Martínez C. (2008). Gibberellins modulate light signaling pathways to prevent *Arabidopsis* seedling de-etiolation in darkness. *Plant J.* 53 324–335. 10.1111/j.1365-313X.2007.03346.x 18053005

[B2] Alabadí D. Gil J. Blázquez M. A. García-Martínez J. L. (2004). Gibberellins repress photomorphogenesis in darkness. *Plant Physiol.* 134 1050–1057. 10.1104/pp.103.035451 14963246 PMC389929

[B3] Baert J.-L. Monte D. Verreman K. Degerny C. Coutte L. de Launoit Y. (2010). The E3 ubiquitin ligase complex component COP1 regulates PEA3 group member stability and transcriptional activity. *Oncogene* 29 1810–1820. 10.1038/onc.2009.471 20062082

[B4] Balcerowicz M. Fittinghoff K. Wirthmueller L. Maier A. Fackendahl P. Fiene G. (2011). Light exposure of *Arabidopsis* seedlings causes rapid de-stabilization as well as selective post-translational inactivation of the repressor of photomorphogenesis SPA2. *Plant J.* 65 712–723. 10.1111/j.1365-313X.2010.04456.x 21235648

[B5] Balcerowicz M. Kerner K. Schenkel C. Hoecker U. (2017). SPA proteins affect the subcellular localization of COP1 in the COP1/SPA ubiquitin ligase complex during photomorphogenesis. *Plant Physiol.* 174 1314–1321. 10.1104/pp.17.00488 28536102 PMC5490927

[B6] Bauer D. Viczián A. Kircher S. Nobis T. Nitschke R. Kunkel T. (2004). Constitutive photomorphogenesis 1 and multiple photoreceptors control degradation of phytochrome interacting factor 3, a transcription factor required for light signaling in *Arabidopsis*. *Plant Cell* 16 1433–1445. 10.1105/tpc.021568 15155879 PMC490037

[B7] Berkel K. van Boer R. J. de Scheres B. ten Tusscher K. (2013). Polar auxin transport: models and mechanisms. *Development* 140 2253–2268. 10.1242/dev.079111 23674599

[B8] Bernardo-García S. de Lucas M. Martínez C. Espinosa-Ruiz A. Davière J.-M. Prat S. (2014). BR-dependent phosphorylation modulates PIF4 transcriptional activity and shapes diurnal hypocotyl growth. *Genes Dev.* 28 1681–1694. 10.1101/gad.243675.114 25085420 PMC4117943

[B9] Bhatnagar A. Singh S. Khurana J. P. Burman N. (2020). HY5-COP1: the central module of light signaling pathway. *J. Plant Biochem. Biotechnol.* 29 590–610. 10.1007/s13562-020-00623-3

[B10] Bianchi E. Denti S. Catena R. Rossetti G. Polo S. Gasparian S. (2003). Characterization of human constitutive photomorphogenesis protein 1, a RING finger ubiquitin ligase that interacts with jun transcription factors and modulates their transcriptional activity. *J. Biol. Chem.* 278 19682–19690. 10.1074/jbc.M212681200 12615916

[B11] Blanco-Touriñán N. Legris M. Minguet E. G. Costigliolo-Rojas C. Nohales M. A. Iniesto E. (2020a). COP1 destabilizes DELLA proteins in *Arabidopsis*. *PNAS* 117 13792–13799. 10.1073/pnas.1907969117 32471952 PMC7306988

[B12] Blanco-Touriñán N. Serrano-Mislata A. Alabadí D. (2020b). Regulation of DELLA proteins by post-translational modifications. *Plant Cell Physiol.* 61 1891–1901. 10.1093/pcp/pcaa113 32886774 PMC7758031

[B13] Burko Y. Seluzicki A. Zander M. Pedmale U. V. Ecker J. R. Chory J. (2020). Chimeric activators and repressors define HY5 activity and reveal a light-regulated feedback mechanism[OPEN]. *Plant Cell* 32 967–983. 10.1105/tpc.19.00772 32086365 PMC7145465

[B14] Bursch K. Toledo-Ortiz G. Pireyre M. Lohr M. Braatz C. Johansson H. (2020). Identification of BBX proteins as rate-limiting cofactors of HY5. *Nat. Plants* 6 921–928. 10.1038/s41477-020-0725-0 32661279

[B15] Cañibano E. Bourbousse C. Garcia-Leon M. Wolff L. Garcia-Baudino C. Barneche F. (2020). DET1-mediated COP1 regulation avoids HY5 activity over second-site targets to tune plant photomorphogenesis. *bioRxiv* [Preprint]. 10.1101/2020.09.30.318253 33711490

[B16] Carrero Z. I. Kollareddy M. Chauhan K. M. Ramakrishnan G. Martinez L. A. (2016). Mutant p53 protects ETS2 from non-canonical COP1/DET1 dependent degradation. *Oncotarget* 7 12554–12567. 10.18632/oncotarget.7275 26871468 PMC4914304

[B17] Catalá R. Medina J. Salinas J. (2011). Integration of low temperature and light signaling during cold acclimation response in *Arabidopsis*. *PNAS* 108 16475–16480. 10.1073/pnas.1107161108 21930922 PMC3182711

[B18] Chang C.-S. J. Maloof J. N. Wu S.-H. (2011). COP1-mediated degradation of BBX22/LZF1 optimizes seedling development in *Arabidopsis*. *Plant Physiol.* 156 228–239. 10.1104/pp.111.175042 21427283 PMC3091042

[B19] Chen G.-H. Liu M.-J. Xiong Y. Sheen J. Wu S.-H. (2018). TOR and RPS6 transmit light signals to enhance protein translation in deetiolating *Arabidopsis* seedlings. *PNAS* 115 12823–12828. 10.1073/pnas.1809526115 30482859 PMC6294885

[B20] Chen H.-J. Fu T.-Y. Yang S.-L. Hsieh H.-L. (2018). FIN219/JAR1 and cryptochrome1 antagonize each other to modulate photomorphogenesis under blue light in *Arabidopsis*. *PLoS Genet.* 14:e1007248. 10.1371/journal.pgen.1007248 29561841 PMC5880400

[B21] Chen H. Huang X. Gusmaroli G. Terzaghi W. Lau O. S. Yanagawa Y. (2010). *Arabidopsis* CULLIN4-damaged DNA binding protein 1 interacts with constitutively photomorphogenic1-suppressor of phya complexes to regulate photomorphogenesis and flowering time. *Plant Cell* 22 108–123. 10.1105/tpc.109.065490 20061554 PMC2828697

[B22] Chen H. Shen Y. Tang X. Yu L. Wang J. Guo L. (2006). *Arabidopsis* CULLIN4 forms an E3 ubiquitin ligase with RBX1 and the CDD complex in mediating light control of development. *Plant Cell* 18 1991–2004. 10.1105/tpc.106.043224 16844902 PMC1533989

[B23] Chen Q. Bai L. Wang W. Shi H. Botella J. R. Zhan Q. (2020). COP1 promotes ABA-induced stomatal closure by modulating the abundance of ABI/HAB and AHG3 phosphatases. *New Phytol.* 229 2035–2049. 10.1111/nph.17001 33048351 PMC7898331

[B24] Chen S. Lory N. Stauber J. Hoecker U. (2015). Photoreceptor specificity in the light-induced and COP1-mediated rapid degradation of the repressor of photomorphogenesis SPA2 in *Arabidopsis*. *PLoS Genet.* 11:e1005516. 10.1371/journal.pgen.1005516 26368289 PMC4569408

[B25] Chen S. Wirthmueller L. Stauber J. Lory N. Holtkotte X. Leson L. (2016). The functional divergence between SPA1 and SPA2 in *Arabidopsis* photomorphogenesis maps primarily to the respective N-terminal kinase-like domain. *BMC Plant Biol.* 16:165. 10.1186/s12870-016-0854-9 27444995 PMC4957354

[B26] Cheng M.-C. Enderle B. Kathare P. K. Islam R. Hiltbrunner A. Huq E. (2020). PCH1 and PCHL directly interact with PIF1, promote its degradation, and inhibit its transcriptional function during photomorphogenesis. *Mol. Plant* 13 499–514. 10.1016/j.molp.2020.02.003 32061894 PMC7167218

[B27] Chico J.-M. Fernández-Barbero G. Chini A. Fernández-Calvo P. Díez-Díaz M. Solano R. (2014). Repression of jasmonate-dependent defenses by shade involves differential regulation of protein stability of MYC transcription factors and their JAZ repressors in *Arabidopsis*. *Plant Cell* 26 1967–1980. 10.1105/tpc.114.125047 24824488 PMC4079362

[B28] Cho S. K. Chaabane S. B. Shah P. Poulsen C. P. Yang S. W. (2014). COP1 E3 ligase protects HYL1 to retain microRNA biogenesis. *Nat. Commun.* 5:5867. 10.1038/ncomms6867 25532508

[B29] Choi C. M. Gray W. M. Mooney S. Hellmann H. (2014). Composition, roles, and regulation of cullin-based ubiquitin E3 ligases. *Arabidopsis Book* 6:e0175. 10.1199/tab.0175 25505853 PMC4262284

[B30] Choi H. H. Guma S. Fang L. Phan L. Ivan C. Baggerly K. (2015). Regulating the stability and localization of CDK inhibitor p27Kip1 via CSN6-COP1 axis. *Cell Cycle* 14 2265–2273. 10.1080/15384101.2015.1046655 25945542 PMC4613181

[B31] Crawford A. J. McLachlan D. H. Hetherington A. M. Franklin K. A. (2012). High temperature exposure increases plant cooling capacity. *Curr. Biol.* 22 R396–R397. 10.1016/j.cub.2012.03.044 22625853

[B32] Crocco C. D. Holm M. Yanovsky M. J. Botto J. F. (2010). AtBBX21 and COP1 genetically interact in the regulation of shade avoidance. *Plant J.* 64 551–562. 10.1111/j.1365-313X.2010.04360.x 21070414

[B33] Datta S. Hettiarachchi C. Johansson H. Holm M. (2007). SALT TOLERANCE HOMOLOG2, a B-box protein in *Arabidopsis* that activates transcription and positively regulates light-mediated development. *Plant Cell* 19 3242–3255. 10.1105/tpc.107.054791 17965270 PMC2174709

[B34] Datta S. Johansson H. Hettiarachchi C. Irigoyen M. L. Desai M. Rubio V. (2008). LZF1/SALT TOLERANCE HOMOLOG3, an *Arabidopsis* B-box protein involved in light-dependent development and gene expression, undergoes COP1-mediated ubiquitination. *Plant Cell* 20 2324–2338. 10.1105/tpc.108.061747 18796637 PMC2570732

[B35] Debrieux D. Trevisan M. Fankhauser C. (2013). Conditional involvement of constitutive photomorphogenic1 in the degradation of phytochrome A. *Plant Physiol.* 161 2136–2145. 10.1104/pp.112.213280 23391578 PMC3613482

[B36] Delker C. Sonntag L. James G. V. Janitza P. Ibañez C. Ziermann H. (2014). The DET1-COP1-HY5 pathway constitutes a multipurpose signaling module regulating plant photomorphogenesis and thermomorphogenesis. *Cell Rep.* 9 1983–1989. 10.1016/j.celrep.2014.11.043 25533339

[B37] Deng X.-W. Caspar T. Quail P. H. (1991). copl: a regulatory locus involved m hght-controlled development and gene expression in *Arabidopsis*. *Genes Dev.* 5 1172–1182. 2065972 10.1101/gad.5.7.1172

[B38] Deng X.-W. Quail P. H. (1992). Genetic and phenotypic characterization of cop1 mutants of *Arabidopsis thaliana*. *Plant J.* 2 83–95. 10.1111/j.1365-313X.1992.00083.x

[B39] Dill A. Thomas S. G. Hu J. Steber C. M. Sun T. (2004). The *Arabidopsis* F-Box protein SLEEPY1 targets gibberellin signaling repressors for gibberellin-induced degradation. *Plant Cell* 16 1392–1405. 10.1105/tpc.020958 15155881 PMC490034

[B40] Dornan D. Shimizu H. Mah A. Dudhela T. Eby M. O’Rourke K. (2006). ATM engages autodegradation of the E3 ubiquitin ligase COP1 after DNA damage. *Science* 313 1122–1126. 10.1126/science.1127335 16931761

[B41] Dornan D. Wertz I. Shimizu H. Arnott D. Frantz G. D. Dowd P. (2004). The ubiquitin ligase COP1 is a critical negative regulator of p53. *Nature* 429 86–92. 10.1038/nature02514 15103385

[B42] Duek P. D. Elmer M. V. van Oosten V. R. Fankhauser C. (2004). The Degradation of HFR1, a Putative bHLH class transcription factor involved in light signaling, is regulated by phosphorylation and requires COP1. *Curr. Biol.* 14 2296–2301. 10.1016/j.cub.2004.12.026 15620659

[B43] Dyachok J. Zhu L. Liao F. He J. Huq E. Blancaflor E. B. (2011). SCAR mediates light-induced root elongation in *Arabidopsis* through photoreceptors and proteasomes. *Plant Cell* 23 3610–3626. 10.1105/tpc.111.088823 21972261 PMC3229138

[B44] Eyers P. A. Keeshan K. Kannan N. (2017). Tribbles in the 21st century: the evolving roles of tribbles pseudokinases in biology and disease. *Trends Cell Biol.* 27 284–298. 10.1016/j.tcb.2016.11.002 27908682 PMC5382568

[B45] Fan X.-Y. Sun Y. Cao D.-M. Bai M.-Y. Luo X.-M. Yang H.-J. (2012). BZS1, a B-box protein, promotes photomorphogenesis downstream of both brassinosteroid and light signaling pathways. *Mol. Plant* 5 591–600. 10.1093/mp/sss041 22535582 PMC3709415

[B46] Favory J.-J. Stec A. Gruber H. Rizzini L. Oravecz A. Funk M. (2009). Interaction of COP1 and UVR8 regulates UV-B-induced photomorphogenesis and stress acclimation in *Arabidopsis*. *EMBO J.* 28 591–601. 10.1038/emboj.2009.4 19165148 PMC2657586

[B47] Fiorucci A.-S. Fankhauser C. (2017). Plant strategies for enhancing access to sunlight. *Curr. Biol.* 27 R931–R940. 10.1016/j.cub.2017.05.085 28898666

[B48] Fittinghoff K. Laubinger S. Nixdorf M. Fackendahl P. Baumgardt R.-L. Batschauer A. (2006). Functional and expression analysis of *Arabidopsis* SPA genes during seedling photomorphogenesis and adult growth. *Plant J.* 47 577–590. 10.1111/j.1365-313X.2006.02812.x 16813571

[B49] Fonseca S. Rubio V. (2019). *Arabidopsis* CRL4 complexes: surveying chromatin states and gene expression. *Front. Plant Sci.* 10:1095. 10.3389/fpls.2019.01095 31608079 PMC6761389

[B50] Gangappa S. N. Botto J. F. (2014). The BBX family of plant transcription factors. *Trends Plant Sci.* 19 460–470. 10.1016/j.tplants.2014.01.010 24582145

[B51] Gangappa S. N. Crocco C. D. Johansson H. Datta S. Hettiarachchi C. Holm M. (2013). The *Arabidopsis* B-BOX protein BBX25 interacts with HY5, negatively regulating BBX22 expression to suppress seedling photomorphogenesis. *Plant Cell* 25 1243–1257. 10.1105/tpc.113.109751 23624715 PMC3663265

[B52] Gangappa S. N. Kumar S. V. (2017). DET1 and HY5 control PIF4-mediated thermosensory elongation growth through distinct mechanisms. *Cell Rep.* 18 344–351. 10.1016/j.celrep.2016.12.046 28076780 PMC5263232

[B53] Gangappa S. N. Prasad V. B. R. Chattopadhyay S. (2010). Functional interconnection of MYC2 and SPA1 in the photomorphogenic seedling development of *Arabidopsis*. *Plant Physiol.* 154 1210–1219. 10.1104/pp.110.163717 20864543 PMC2971600

[B54] Gupta N. Nath U. (2020). Integration of light and hormone response during seedling establishment. *J. Plant Biochem. Biotechnol.* 29 652–664. 10.1007/s13562-020-00628-y

[B55] Han X. Huang X. Deng X. W. (2020). The photomorphogenic central repressor COP1: conservation and functional diversification during evolution. *Plant Commun.* 1:100044. 10.1016/j.xplc.2020.100044 33367240 PMC7748024

[B56] Hayes S. Sharma A. Fraser D. P. Trevisan M. Cragg-Barber C. K. Tavridou E. (2017). UV-B perceived by the UVR8 photoreceptor inhibits plant thermomorphogenesis. *Curr. Biol.* 27 120–127. 10.1016/j.cub.2016.11.004 27989670 PMC5226890

[B57] He J.-X. Gendron J. M. Yang Y. Li J. Wang Z.-Y. (2002). The GSK3-like kinase BIN2 phosphorylates and destabilizes BZR1, a positive regulator of the brassinosteroid signaling pathway in *Arabidopsis*. *PNAS* 99 10185–10190. 10.1073/pnas.152342599 12114546 PMC126645

[B58] Heng Y. Jiang Y. Zhao X. Zhou H. Wang X. Deng X. W. (2019). BBX4, a phyB-interacting and modulated regulator, directly interacts with PIF3 to fine tune red light-mediated photomorphogenesis. *PNAS* 116 26049–26056. 10.1073/pnas.1915149116 31776262 PMC6925995

[B59] Hoecker U. (2017). The activities of the E3 ubiquitin ligase COP1/SPA, a key repressor in light signaling. *Curr. Opin. Plant Biol.* 37 63–69. 10.1016/j.pbi.2017.03.015 28433946

[B60] Hoecker U. Quail P. H. (2001). The phytochrome A-specific signaling intermediate SPA1 interacts directly with COP1, a constitutive repressor of light signaling in *Arabidopsis*. *J. Biol. Chem.* 276 38173–38178. 10.1074/jbc.M103140200 11461903

[B61] Hoecker U. Tepperman J. M. Quail P. H. (1999). SPA1, a WD-repeat protein specific to phytochrome a signal transduction. *Science* 284 496–499. 10.1126/science.284.5413.496 10205059

[B62] Holm M. Hardtke C. S. Gaudet R. Deng X.-W. (2001). Identification of a structural motif that confers specific interaction with the WD40 repeat domain of *Arabidopsis* COP1. *EMBO J.* 20 118–127. 10.1093/emboj/20.1.118 11226162 PMC140188

[B63] Holm M. Ma L.-G. Qu L.-J. Deng X.-W. (2002). Two interacting bZIP proteins are direct targets of COP1-mediated control of light-dependent gene expression in *Arabidopsis*. *Genes Dev.* 16 1247–1259. 10.1101/gad.969702 12023303 PMC186273

[B64] Holtkotte X. Dieterle S. Kokkelink L. Artz O. Leson L. Fittinghoff K. (2016). Mutations in the N-terminal kinase-like domain of the repressor of photomorphogenesis SPA1 severely impair SPA1 function but not light responsiveness in *Arabidopsis*. *Plant J.* 88 205–218. 10.1111/tpj.13241 27310313

[B65] Holtkotte X. Ponnu J. Ahmad M. Hoecker U. (2017). The blue light-induced interaction of cryptochrome 1 with COP1 requires SPA proteins during *Arabidopsis* light signaling. *PLoS Genet.* 13:e1007044. 10.1371/journal.pgen.1007044 28991901 PMC5648270

[B66] Hong S. H. Kim H. J. Ryu J. S. Choi H. Jeong S. Shin J. (2008). CRY1 inhibits COP1-mediated degradation of BIT1, a MYB transcription factor, to activate blue light-dependent gene expression in *Arabidopsis*. *Plant J.* 55 361–371. 10.1111/j.1365-313X.2008.03508.x 18397371

[B67] Hsieh H.-L. Okamoto H. (2014). Molecular interaction of jasmonate and phytochrome A signalling. *J. Exp. Bot.* 65 2847–2857. 10.1093/jxb/eru230 24868039

[B68] Hua Z. Vierstra R. D. (2011). The cullin-RING ubiquitin-protein ligases. *Annu. Rev. Plant Biol.* 62 299–334. 10.1146/annurev-arplant-042809-112256 21370976

[B69] Huang H. Liu B. Liu L. Song S. (2017). Jasmonate action in plant growth and development. *J. Exp. Bot.* 68 1349–1359. 10.1093/jxb/erw495 28158849

[B70] Iglesias M. J. Sellaro R. Zurbriggen M. D. Casal J. J. (2018). Multiple links between shade avoidance and auxin networks. *J Exp Bot.* 69 213–228. 10.1093/jxb/erx295 29036463

[B71] Irigoyen M. L. Iniesto E. Rodriguez L. Puga M. I. Yanagawa Y. Pick E. (2014). Targeted degradation of abscisic acid receptors is mediated by the ubiquitin ligase substrate adaptor DDA1 in *Arabidopsis*. *Plant Cell* 26 712–728. 10.1105/tpc.113.122234 24563205 PMC3967035

[B72] Jang I.-C. Henriques R. Seo H. S. Nagatani A. Chua N.-H. (2010). *Arabidopsis* phytochrome interacting factor proteins promote phytochrome B polyubiquitination by COP1 E3 ligase in the nucleus. *Plant Cell* 22 2370–2383. 10.1105/tpc.109.072520 20605855 PMC2929111

[B73] Jang I.-C. Yang J.-Y. Seo H. S. Chua N.-H. (2005). HFR1 is targeted by COP1 E3 ligase for post-translational proteolysis during phytochrome A signaling. *Genes Dev.* 19 593–602. 10.1101/gad.1247205 15741320 PMC551579

[B74] Jang K. Gil Lee H. Jung S.-J. Paek N.-C. Joon Seo P. (2015). The E3 Ubiquitin Ligase COP1 Regulates Thermosensory Flowering by Triggering GI Degradation in *Arabidopsis*. *Sci. Rep.* 5:12071. 10.1038/srep12071 26159740 PMC4498190

[B75] Jang S. Marchal V. Panigrahi K. C. S. Wenkel S. Soppe W. Deng X.-W. (2008). *Arabidopsis* COP1 shapes the temporal pattern of CO accumulation conferring a photoperiodic flowering response. *EMBO J.* 27 1277–1288. 10.1038/emboj.2008.68 18388858 PMC2291449

[B76] Jeong R.-D. Chandra-Shekara A. C. Barman S. R. Navarre D. Klessig D. F. Kachroo A. (2010). Cryptochrome 2 and phototropin 2 regulate resistance protein-mediated viral defense by negatively regulating an E3 ubiquitin ligase. *Proc. Natl. Acad. Sci. U.S.A.* 107 13538–13543. 10.1073/pnas.1004529107 20624951 PMC2922132

[B77] Jia K.-P. Luo Q. He S.-B. Lu X.-D. Yang H.-Q. (2014). Strigolactone-regulated hypocotyl elongation is dependent on cryptochrome and phytochrome signaling pathways in *Arabidopsis*. *Mol. Plant* 7 528–540. 10.1093/mp/sst093 24126495

[B78] Jiang L. Wang Y. Li Q.-F. Björn L. O. He J.-X. Li S.-S. (2012). *Arabidopsis* STO/BBX24 negatively regulates UV-B signaling by interacting with COP1 and repressing HY5 transcriptional activity. *Cell Res.* 22:1046. 10.1038/cr.2012.34 22410790 PMC3367526

[B79] Jung J.-H. Domijan M. Klose C. Biswas S. Ezer D. Gao M. (2016). Phytochromes function as thermosensors in *Arabidopsis*. *Science* 354 886–889. 10.1126/science.aaf6005 27789797

[B80] Kahle N. Sheerin D. J. Fischbach P. Koch L.-A. Schwenk P. Lambert D. (2020). COLD REGULATED 27 and 28 are targets of CONSTITUTIVELY PHOTOMORPHOGENIC 1 and negatively affect phytochrome B signalling. *Plant J.* 104 1038–1053. 10.1111/tpj.14979 32890447

[B81] Kathare P. K. Xu X. Nguyen A. Huq E. (2020). A COP1-PIF-HEC regulatory module fine-tunes photomorphogenesis in *Arabidopsis*. *Plant J.* 104 113–123. 10.1111/tpj.14908 32652745 PMC7959245

[B82] Kim B. Jeong Y. J. Corvalán C. Fujioka S. Cho S. Park T. (2014). Darkness and gulliver2/phyB mutation decrease the abundance of phosphorylated BZR1 to activate brassinosteroid signaling in *Arabidopsis*. *Plant J.* 77 737–747. 10.1111/tpj.12423 24387668 PMC4282538

[B83] Kim J. Y. Jang I.-C. Seo H. S. (2016). COP1 controls abiotic stress responses by modulating AtSIZ1 function through its E3 ubiquitin ligase activity. *Front. Plant Sci.* 7:1182. 10.3389/fpls.2016.01182 27536318 PMC4971112

[B84] Kim S. Hwang G. Lee S. Zhu J.-Y. Paik I. Nguyen T. T. (2017). High ambient temperature represses anthocyanin biosynthesis through degradation of HY5. *Front. Plant Sci.* 8:1787. 10.3389/fpls.2017.01787 29104579 PMC5655971

[B85] Kung J. E. Jura N. (2019). The pseudokinase TRIB1 toggles an intramolecular switch to regulate COP1 nuclear export. *EMBO J.* 38:e99708. 10.15252/embj.201899708 30692133 PMC6376274

[B86] Lau K. Podolec R. Chappuis R. Ulm R. Hothorn M. (2019). Plant photoreceptors and their signaling components compete for COP1 binding via VP peptide motifs. *EMBO J.* 10:e102140. 10.15252/embj.2019102140 31304983 PMC6745501

[B87] Lau O. S. Deng X. W. (2012). The photomorphogenic repressors COP1 and DET1: 20 years later. *Trends Plant Sci.* 17 584–593. 10.1016/j.tplants.2012.05.004 22705257

[B88] Laubinger S. Fittinghoff K. Hoecker U. (2004). The SPA quartet: a family of WD-repeat proteins with a central role in suppression of photomorphogenesis in *Arabidopsis*. *Plant Cell* 16 2293–2306. 10.1105/tpc.104.024216 15308756 PMC520934

[B89] Laubinger S. Marchal V. Gentilhomme J. Wenkel S. Adrian J. Jang S. (2006). *Arabidopsis* SPA proteins regulate photoperiodic flowering and interact with the floral inducer CONSTANS to regulate its stability. *Development* 133 3213–3222. 10.1242/dev.02481 16854975

[B90] Lee J.-H. Jung J.-H. Park C.-M. (2017). Light inhibits COP1-mediated degradation of ICE transcription factors to induce stomatal development in *Arabidopsis*. *Plant Cell* 29 2817–2830. 10.1105/tpc.17.00371 29070509 PMC5728130

[B91] Lee S. Paik I. Huq E. (2020). SPAs promote thermomorphogenesis by regulating the phyB-PIF4 module in *Arabidopsis*. *Development* 147:dev189233. 10.1242/dev.189233 32994167 PMC7561471

[B92] Lee Y.-H. Andersen J. B. Song H.-T. Judge A. D. Seo D. Ishikawa T. (2010). Definition of ubiquitination modulator COP1 as a novel therapeutic target in human hepatocellular carcinoma. *Cancer Res.* 70 8264–8269. 10.1158/0008-5472.CAN-10-0749 20959491 PMC2970744

[B93] Legris M. Klose C. Burgie E. S. Rojas C. C. R. Neme M. Hiltbrunner A. (2016). Phytochrome B integrates light and temperature signals in *Arabidopsis*. *Science* 354 897–900. 10.1126/science.aaf5656 27789798

[B94] Legris M. Nieto C. Sellaro R. Prat S. Casal J. J. (2017). Perception and signalling of light and temperature cues in plants. *Plant J.* 90 683–697. 10.1111/tpj.13467 28008680

[B95] Leivar P. Monte E. Al-Sady B. Carle C. Storer A. Alonso J. M. (2008). The *Arabidopsis* phytochrome-interacting factor PIF7, together with PIF3 and PIF4, regulates responses to prolonged red light by modulating phyB levels. *Plant Cell* 20 337–352. 10.1105/tpc.107.052142 18252845 PMC2276449

[B96] Li J. Nagpal P. Vitart V. McMorris T. C. Chory J. (1996). A role for brassinosteroids in light-dependent development of *Arabidopsis*. *Science* 272 398–401. 10.1126/science.272.5260.398 8602526

[B97] Li X. Liu C. Zhao Z. Ma D. Zhang J. Yang Y. (2020). COR27 and COR28 are novel regulators of the COP1–HY5 regulatory hub and photomorphogenesis in *Arabidopsis*. *Plant Cell* 32 3139–3154. 10.1105/tpc.20.00195 32769132 PMC7534460

[B98] Lian H.-L. He S.-B. Zhang Y.-C. Zhu D.-M. Zhang J.-Y. Jia K.-P. (2011). Blue-light-dependent interaction of cryptochrome 1 with SPA1 defines a dynamic signaling mechanism. *Genes Dev.* 25 1023–1028. 10.1101/gad.2025111 21511872 PMC3093117

[B99] Lian N. Liu X. Wang X. Zhou Y. Li H. Li J. (2017). COP1 mediates dark-specific degradation of microtubule-associated protein WDL3 in regulating *Arabidopsis* hypocotyl elongation. *PNAS* 114 12321–12326. 10.1073/pnas.1708087114 29087315 PMC5699047

[B100] Liang T. Yang Y. Liu H. (2019). Signal transduction mediated by the plant UV-B photoreceptor UVR8. *New Phytol.* 221 1247–1252. 10.1111/nph.15469 30315741

[B101] Lim G.-H. Hoey T. Zhu S. Clavel M. Yu K. Navarre D. (2018). COP1, a negative regulator of photomorphogenesis, positively regulates plant disease resistance via double-stranded RNA binding proteins. *PLoS Pathog.* 14:e1006894. 10.1371/journal.ppat.1006894 29513740 PMC5871017

[B102] Lin F. Jiang Y. Li J. Yan T. Fan L. Liang J. (2018). B-Box Domain Protein28 negatively regulates photomorphogenesis by repressing the activity of transcription factor HY5 and undergoes COP1-mediated degradation. *Plant Cell* 30 2006–2019. 10.1105/tpc.18.00226 30099385 PMC6181009

[B103] Lin F. Xu D. Jiang Y. Chen H. Fan L. Holm M. (2017). Phosphorylation and negative regulation of CONSTITUTIVELY PHOTOMORPHOGENIC 1 by PINOID in *Arabidopsis*. *PNAS* 2017:201702984. 10.1073/pnas.1702984114 28584104 PMC5488946

[B104] Lin X.-L. Niu D. Hu Z.-L. Kim D. H. Jin Y. H. Cai B. (2016). An *Arabidopsis* SUMO E3 Ligase, SIZ1, negatively regulates photomorphogenesis by promoting COP1 activity. *PLoS Genet.* 12:e1006016. 10.1371/journal.pgen.1006016 27128446 PMC4851335

[B105] Ling J.-J. Li J. Zhu D. Deng X. W. (2017). Noncanonical role of *Arabidopsis* COP1/SPA complex in repressing BIN2-mediated PIF3 phosphorylation and degradation in darkness. *PNAS* 2017:201700850. 10.1073/pnas.1700850114 28292892 PMC5380025

[B106] Liu B. Long H. Yan J. Ye L. Zhang Q. Chen H. (2020). A HY5-COL3-COL13 regulatory chain for controlling hypocotyl elongation in *Arabidopsis*. *Plant Cell Environ.* 44 130–142. 10.1111/pce.13899 33011994

[B107] Liu B. Zuo Z. Liu H. Liu X. Lin C. (2011). *Arabidopsis* cryptochrome 1 interacts with SPA1 to suppress COP1 activity in response to blue light. *Genes Dev.* 25 1029–1034. 10.1101/gad.2025011 21511871 PMC3093118

[B108] Liu L.-J. Zhang Y.-C. Li Q.-H. Sang Y. Mao J. Lian H.-L. (2008). COP1-mediated ubiquitination of CONSTANS is implicated in cryptochrome regulation of flowering in *Arabidopsis*. *Plant Cell* 20 292–306. 10.1105/tpc.107.057281 18296627 PMC2276438

[B109] Liu Q. Su T. He W. Ren H. Liu S. Chen Y. (2020). Photooligomerization determines photosensitivity and photoreactivity of plant cryptochromes. *Mol. Plant* 13 398–413. 10.1016/j.molp.2020.01.002 31953223 PMC7056577

[B110] Liu Q. Wang Q. Liu B. Wang W. Wang X. Park J. (2016). The blue light-dependent polyubiquitination and degradation of *Arabidopsis* cryptochrome2 requires multiple E3 ubiquitin ligases. *Plant Cell Physiol.* 57 2175–2186. 10.1093/pcp/pcw134 27516416 PMC6083963

[B111] Lopez-Molina L. Mongrand S. Kinoshita N. Chua N.-H. (2003). AFP is a novel negative regulator of ABA signaling that promotes ABI5 protein degradation. *Genes Dev.* 17 410–418. 10.1101/gad.1055803 12569131 PMC195991

[B112] Lu X.-D. Zhou C.-M. Xu P.-B. Luo Q. Lian H.-L. Yang H.-Q. (2015). Red-light-dependent interaction of phyB with SPA1 promotes COP1–SPA1 dissociation and photomorphogenic development in *Arabidopsis*. *Mol. Plant* 8 467–478. 10.1016/j.molp.2014.11.025 25744387

[B113] Luo Q. Lian H.-L. He S.-B. Li L. Jia K.-P. Yang H.-Q. (2014). COP1 and phyB physically interact with PIL1 to regulate its stability and photomorphogenic development in *Arabidopsis*. *Plant Cell* 26 2441–2456. 10.1105/tpc.113.121657 24951480 PMC4114944

[B114] Luo X.-M. Lin W.-H. Zhu S. Zhu J.-Y. Sun Y. Fan X.-Y. (2010). Integration of light- and brassinosteroid-signaling pathways by a GATA transcription factor in *Arabidopsis*. *Dev. Cell* 19 872–883. 10.1016/j.devcel.2010.10.023 21145502 PMC3022420

[B115] Ma L. Zhao H. Deng X. W. (2003). Analysis of the mutational effects of the COP/DET/FUS loci on genome expression profiles reveals their overlapping yet not identical roles in regulating *Arabidopsis* seedling development. *Development* 130 969–981. 10.1242/dev.00281 12538522

[B116] Maier A. Hoecker U. (2015). COP1/SPA ubiquitin ligase complexes repress anthocyanin accumulation under low light and high light conditions. *Plant Signal. Behav.* 10:e970440. 10.4161/15592316.2014.970440 25482806 PMC4622049

[B117] Maier A. Schrader A. Kokkelink L. Falke C. Welter B. Iniesto E. (2013). Light and the E3 ubiquitin ligase COP1/SPA control the protein stability of the MYB transcription factors PAP1 and PAP2 involved in anthocyanin accumulation in *Arabidopsis*. *Plant J.* 74 638–651. 10.1111/tpj.12153 23425305

[B118] Martínez C. Nieto C. Prat S. (2018). Convergent regulation of PIFs and the E3 ligase COP1/SPA1 mediates thermosensory hypocotyl elongation by plant phytochromes. *Curr. Opin. Plant Biol.* 45 188–203. 10.1016/j.pbi.2018.09.006 30273926

[B119] Matsui K. Umemura Y. Ohme-Takagi M. (2008). AtMYBL2, a protein with a single MYB domain, acts as a negative regulator of anthocyanin biosynthesis in *Arabidopsis*. *Plant J.* 55 954–967. 10.1111/j.1365-313X.2008.03565.x 18532977

[B120] Matsui M. Stoop C. D. von Arnim A. G. Wei N. Deng X. W. (1995). *Arabidopsis* COP1 protein specifically interacts in vitro with a cytoskeleton-associated protein. *CIP1. PNAS* 92 4239–4243. 10.1073/pnas.92.10.4239 7753789 PMC41919

[B121] Mayer R. Raventos D. Chua N. H. (1996). det1, cop1, and cop9 mutations cause inappropriate expression of several gene sets. *Plant Cell* 8 1951–1959. 10.1105/tpc.8.11.1951 8953766 PMC161326

[B122] Mazzella M. A. Casal J. J. Muschietti J. P. Fox A. R. (2014). Hormonal networks involved in apical hook development in darkness and their response to light. *Front. Plant Sci.* 5:52. 10.3389/fpls.2014.00052 24616725 PMC3935338

[B123] Mazzucotelli E. Belloni S. Marone D. De Leonardis A. Guerra D. Di Fonzo N. (2006). The E3 ubiquitin ligase gene family in plants: regulation by degradation. *Curr. Genom.* 7 509–522. 10.2174/138920206779315728 18369404 PMC2269001

[B124] McNellis T. W. von Arnim A. G. Araki T. Komeda Y. Miséra S. Deng X. W. (1994). Genetic and molecular analysis of an allelic series of cop1 mutants suggests functional roles for the multiple protein domains. *Plant Cell* 6 487–500. 10.1105/tpc.6.4.487 8205001 PMC160452

[B125] Menon C. Sheerin D. J. Hiltbrunner A. (2016). SPA proteins: SPAnning the gap between visible light and gene expression. *Planta* 244 297–312. 10.1007/s00425-016-2509-3 27100111

[B126] Moazzam-Jazi M. Ghasemi S. Seyedi S. M. Niknam V. (2018). COP1 plays a prominent role in drought stress tolerance in *Arabidopsis* and Pea. *Plant Physiol. Biochem.* 130 678–691. 10.1016/j.plaphy.2018.08.015 30139551

[B127] Nakagawa M. Komeda Y. (2004). Flowering of *Arabidopsis* cop1 mutants in darkness. *Plant Cell Physiol.* 45 398–406. 10.1093/pcp/pch047 15111714

[B128] Nam K. H. Li J. (2002). BRI1/BAK1, a receptor kinase pair mediating brassinosteroid signaling. *Cell* 110 203–212. 10.1016/S0092-8674(02)00814-0 12150928

[B129] Ni W. Xu S.-L. Tepperman J. M. Stanley D. J. Maltby D. A. Gross J. D. (2014). A mutually assured destruction mechanism attenuates light signaling in *Arabidopsis*. *Science* 344 1160–1164. 10.1126/science.1250778 24904166 PMC4414656

[B130] Nieto C. Luengo L. M. Prat S. (2020). Regulation of COP1 function by brassinosteroid signaling. *Front. Plant Sci.* 11:1151. 10.3389/fpls.2020.01151 32849709 PMC7411146

[B131] Nixdorf M. Hoecker U. (2010). SPA1 and DET1 act together to control photomorphogenesis throughout plant development. *Planta* 231 825–833. 10.1007/s00425-009-1088-y 20041285

[B132] Nozue K. Tat A. V. Devisetty U. K. Robinson M. Mumbach M. R. Ichihashi Y. (2015). Shade avoidance components and pathways in adult plants revealed by phenotypic profiling. *PLoS Genet.* 11:e1004953. 10.1371/journal.pgen.1004953 25874869 PMC4398415

[B133] Oh J. Park E. Song K. Bae G. Choi G. (2020). PHYTOCHROME INTERACTING FACTOR8 inhibits phytochrome a-mediated far-red light responses in *Arabidopsis*. *Plant Cell* 32 186–205. 10.1105/tpc.19.00515 31732705 PMC6961613

[B134] Oravecz A. Baumann A. Máté Z. Brzezinska A. Molinier J. Oakeley E. J. (2006). CONSTITUTIVELY PHOTOMORPHOGENIC1 is required for the UV-B response in *Arabidopsis*. *Plant Cell* 18 1975–1990. 10.1105/tpc.105.040097 16829591 PMC1533968

[B135] Ordoñez-Herrera N. Fackendahl P. Yu X. Schaefer S. Koncz C. Hoecker U. (2015). A cop1 spa mutant deficient in COP1 and SPA proteins reveals partial co-action of COP1 and SPA during *Arabidopsis* post-embryonic development and photomorphogenesis. *Mol. Plant* 8 479–481. 10.1016/j.molp.2014.11.026 25667004

[B136] Ordoñez-Herrera N. Trimborn L. Menje M. Henschel M. Robers L. Kaufholdt D. (2018). The transcription factor COL12 is a substrate of the COP1/SPA E3 ligase and regulates flowering time and plant architecture1. *Plant Physiol.* 176 1327–1340. 10.1104/pp.17.01207 29187570 PMC5813546

[B137] Osterlund M. T. Hardtke C. S. Wei N. Deng X. W. (2000). Targeted destabilization of HY5 during light-regulated development of *Arabidopsis*. *Nature* 405:462. 10.1038/35013076 10839542

[B138] Pacín M. Legris M. Casal J. J. (2013). COP1 re-accumulates in the nucleus under shade. *Plant J.* 75 631–641. 10.1111/tpj.12226 23647163

[B139] Pacín M. Legris M. Casal J. J. (2014). Rapid decline in nuclear COSTITUTIVE PHOTOMORPHOGENESIS1 abundance anticipates the stabilization of its target ELONGATED HYPOCOTYL5 in the light. *Plant Physiol.* 164 1134–1138. 10.1104/pp.113.234245 24434030 PMC3938608

[B140] Pacín M. Semmoloni M. Legris M. Finlayson S. A. Casal J. J. (2016). Convergence of CONSTITUTIVE PHOTOMORPHOGENESIS 1 and PHYTOCHROME INTERACTING FACTOR signalling during shade avoidance. *New Phytol.* 211 967–979. 10.1111/nph.13965 27105120

[B141] Paik I. Chen F. Pham V. N. Zhu L. Kim J.-I. Huq E. (2019). A phyB-PIF1-SPA1 kinase regulatory complex promotes photomorphogenesis in *Arabidopsis*. *Nat. Commun.* 10 1–17. 10.1038/s41467-019-12110-y 31527679 PMC6746701

[B142] Palayam M. Ganapathy J. Guercio A. M. Tal L. Deck S. L. Shabek N. (2021). Structural insights into photoactivation of plant Cryptochrome-2. *Commun. Biol.* 4 1–11. 10.1038/s42003-020-01531-x 33398020 PMC7782693

[B143] Park Y.-J. Lee H.-J. Ha J.-H. Kim J. Y. Park C.-M. (2017). COP1 conveys warm temperature information to hypocotyl thermomorphogenesis. *New Phytol.* 215 269–280. 10.1111/nph.14581 28418582

[B144] Pepper A. Delaney T. Washburnt T. Poole D. Chory J. (1994). DET1, a negative regulator of light-mediated development and gene expression in *Arabidopsis*, encodes a novel nuclear-localized protein. *Cell* 78 109–116. 10.1016/0092-8674(94)90577-0 8033202

[B145] Pfeiffer A. Janocha D. Dong Y. Medzihradszky A. Schöne S. Daum G. (2016). Integration of light and metabolic signals for stem cell activation at the shoot apical meristem. *eLife Sci.* 5:e17023. 10.7554/eLife.17023 27400267 PMC4969040

[B146] Pham V. N. Kathare P. K. Huq E. (2018a). Dynamic regulation of PIF5 by COP1-SPA complex to optimize photomorphogenesis in *Arabidopsis*. *Plant J.* 96 260–273. 10.1111/tpj.14074 30144338 PMC6177295

[B147] Pham V. N. Kathare P. K. Huq E. (2018b). Phytochromes and phytochrome interacting factors. *Plant Physiol.* 176 1025–1038. 10.1104/pp.17.01384 29138351 PMC5813575

[B148] Podolec R. Ulm R. (2018). Photoreceptor-mediated regulation of the COP1/SPA E3 ubiquitin ligase. *Curr. Opin. Plant Biol.* 45 18–25. 10.1016/j.pbi.2018.04.018 29775763

[B149] Ponnu J. (2020). Molecular mechanisms suppressing COP1/SPA E3 ubiquitin ligase activity in blue light. *Physiol. Plant.* 169 418–429. 10.1111/ppl.13103 32248530

[B150] Ponnu J. Riedel T. Penner E. Schrader A. Hoecker U. (2019). Cryptochrome 2 competes with COP1 substrates to repress COP1 ubiquitin ligase activity during *Arabidopsis* photomorphogenesis. *PNAS* 116 27133–27141. 10.1073/pnas.1909181116 31822614 PMC6936435

[B151] Quint M. Delker C. Franklin K. A. Wigge P. A. Halliday K. J. van Zanten M. (2016). Molecular and genetic control of plant thermomorphogenesis. *Nat. Plants* 2:15190. 10.1038/nplants.2015.190 27250752

[B152] Ren C. Zhu X. Zhang P. Gong Q. (2016). *Arabidopsis* COP1-interacting protein 1 is a positive regulator of ABA response. *Biochem. Biophys. Res. Commun.* 477 847–853. 10.1016/j.bbrc.2016.06.147 27372427

[B153] Rizzini L. Levine D. C. Perelis M. Bass J. Peek C. B. Pagano M. (2019). Cryptochromes-mediated inhibition of the CRL4Cop1-complex assembly defines an evolutionary conserved signaling mechanism. *Curr. Biol.* 29 1954.e4–1962.e4. 10.1016/j.cub.2019.04.073 31155351 PMC6581574

[B154] Rolauffs S. Fackendahl P. Sahm J. Fiene G. Hoecker U. (2012). *Arabidopsis* COP1 and SPA genes are essential for plant elongation but not for acceleration of flowering time in response to a low red light to far-red light ratio. *Plant Physiol.* 160 2015–2027. 10.1104/pp.112.207233 23093358 PMC3510128

[B155] Saijo Y. Sullivan J. A. Wang H. Yang J. Shen Y. Rubio V. (2003). The COP1–SPA1 interaction defines a critical step in phytochrome A-mediated regulation of HY5 activity. *Genes Dev.* 17 2642–2647. 10.1101/gad.1122903 14597662 PMC280614

[B156] Saijo Y. Zhu D. Li J. Rubio V. Zhou Z. Shen Y. (2008). *Arabidopsis* COP1/SPA1 complex and FHY1/FHY3 associate with distinct phosphorylated forms of phytochrome A in balancing light signaling. *Mol. Cell.* 31 607–613. 10.1016/j.molcel.2008.08.003 18722184 PMC2597559

[B157] Sarid-Krebs L. Panigrahi K. C. S. Fornara F. Takahashi Y. Hayama R. Jang S. (2015). Phosphorylation of CONSTANS and its COP1-dependent degradation during photoperiodic flowering of *Arabidopsis*. *Plant J.* 84 451–463. 10.1111/tpj.13022 26358558

[B158] Sassi M. Lu Y. Zhang Y. Wang J. Dhonukshe P. Blilou I. (2012). COP1 mediates the coordination of root and shoot growth by light through modulation of PIN1- and PIN2-dependent auxin transport in *Arabidopsis*. *Development* 139 3402–3412. 10.1242/dev.078212 22912415

[B159] Schrader A. Welter B. Hulskamp M. Hoecker U. Uhrig J. F. (2013). MIDGET connects COP1-dependent development with endoreduplication in *Arabidopsis thaliana*. *Plant J.* 75 67–79. 10.1111/tpj.12199 23573936

[B160] Schulman B. A. Carrano A. C. Jeffrey P. D. Bowen Z. Kinnucan E. R. Finnin M. S. (2000). Insights into SCF ubiquitin ligases from the structure of the Skp1-Skp2 complex. *Nature* 408 381–386. 10.1038/35042620 11099048

[B161] Seo H. S. Watanabe E. Tokutomi S. Nagatani A. Chua N.-H. (2004). Photoreceptor ubiquitination by COP1 E3 ligase desensitizes phytochrome A signaling. *Genes Dev.* 18 617–622. 10.1101/gad.1187804 15031264 PMC387237

[B162] Seo H. S. Yang J.-Y. Ishikawa M. Bolle C. Ballesteros M. L. Chua N.-H. (2003). LAF1 ubiquitination by COP1 controls photomorphogenesis and is stimulated by SPA1. *Nature* 423 995–999. 10.1038/nature01696 12827204

[B163] Shao K. Zhang X. Li X. Hao Y. Huang X. Ma M. (2020). The oligomeric structures of plant cryptochromes. *Nat. Struct. Mol. Biol.* 27 480–488. 10.1038/s41594-020-0420-x 32398825

[B164] Sheerin D. J. Menon C. Oven-Krockhaus S. Z. Enderle B. Zhu L. Johnen P. (2015). Light-activated phytochrome A and B interact with members of the SPA family to promote photomorphogenesis in *Arabidopsis* by reorganizing the COP1/SPA complex. *Plant Cell* 27 189–201. 10.1105/tpc.114.134775 25627066 PMC4330587

[B165] Shi H. Liu R. Xue C. Shen X. Wei N. Deng X. W. (2016). Seedlings transduce the depth and mechanical pressure of covering soil using COP1 and ethylene to regulate EBF1/EBF2 for soil emergence. *Curr. Biol.* 26 139–149. 10.1016/j.cub.2015.11.053 26748855 PMC5108888

[B166] Shi Q.-M. Yang X. Song L. Xue H.-W. (2011). *Arabidopsis* MSBP1 is activated by HY5 and HYH and is involved in photomorphogenesis and brassinosteroid sensitivity regulation. *Mol. Plant* 4 1092–1104. 10.1093/mp/ssr049 21715650

[B167] Song L. I Zhou X.-Y. Li L. I Xue L.-J. Yang X. I Xue H.-W. (2009). Genome-wide analysis revealed the complex regulatory network of brassinosteroid effects in photomorphogenesis. *Mol. Plant* 2 755–772. 10.1093/mp/ssp039 19825654

[B168] Song Y. Liu Y. Pan S. Xie S. Wang Z. Zhu X. (2020). Role of the COP1 protein in cancer development and therapy. *Semin. Cancer Biol.* 67 43–52. 10.1016/j.semcancer.2020.02.001 32027978

[B169] Song Z. Yan T. Liu J. Bian Y. Heng Y. Lin F. (2020). BBX28/BBX29, HY5 and BBX30/31 form a feedback loop to fine-tune photomorphogenic development. *Plant J.* 104 377–390. 10.1111/tpj.14929 32654323

[B170] Stacey M. G. Hicks S. N. von Arnim A. G. (1999). Discrete domains mediate the light-responsive nuclear and cytoplasmic localization of *Arabidopsis* COP1. *Plant Cell* 11 349–363. 10.1105/tpc.11.3.349 10072396 PMC144184

[B171] Stacey M. G. Kopp O. R. Kim T.-H. von Arnim A. G. (2000). Modular domain structure of *Arabidopsis* COP1. Reconstitution of activity by fragment complementation and mutational analysis of a nuclear localization signal in planta. *Plant Physiol.* 124 979–990. 10.1104/pp.124.3.979 11080276 PMC59198

[B172] Stoop-Myer C. Torii K. U. McNellis T. W. Coleman J. E. Deng X.-W. (1999). The N-terminal fragment of *Arabidopsis* photomorphogenic repressor COP1 maintains partial function and acts in a concentration-dependent manner. *Plant J.* 20 713–717. 10.1046/j.1365-313X.1999.00639.x 10652143

[B173] Subramanian C. Kim B.-H. Lyssenko N. N. Xu X. Johnson C. H. von Arnim A. G. (2004). The *Arabidopsis* repressor of light signaling, COP1, is regulated by nuclear exclusion: mutational analysis by bioluminescence resonance energy transfer. *PNAS* 101 6798–6802. 10.1073/pnas.0307964101 15084749 PMC404125

[B174] Suzuki G. Yanagawa Y. Kwok S. F. Matsui M. Deng X.-W. (2002). *Arabidopsis* COP10 is a ubiquitin-conjugating enzyme variant that acts together with COP1 and the COP9 signalosome in repressing photomorphogenesis. *Genes Dev.* 16 554–559. 10.1101/gad.964602 11877375 PMC155353

[B175] Swain S. Jiang H.-W. Hsieh H.-L. (2017). FAR-RED INSENSITIVE 219/JAR1 contributes to shade avoidance responses of *Arabidopsis* seedlings by modulating key shade signaling components. *Front. Plant Sci.* 8:1901. 10.3389/fpls.2017.01901 29163619 PMC5673645

[B176] Tavridou E. Pireyre M. Ulm R. (2020). Degradation of the transcription factors PIF4 and PIF5 under UV-B promotes UVR8-mediated hypocotyl growth inhibition in *Arabidopsis*. *Plant J.* 101 507–517. 10.1111/tpj.14556 31571300 PMC7027837

[B177] Torii K. U. McNellis T. W. Deng X. W. (1998). Functional dissection of *Arabidopsis* COP1 reveals specific roles of its three structural modules in light control of seedling development. *EMBO J.* 17 5577–5587. 10.1093/emboj/17.19.5577 9755158 PMC1170886

[B178] Torii K. U. Stoop-Myer C. D. Okamoto H. Coleman J. E. Matsui M. Deng X. W. (1999). The RING finger motif of photomorphogenic repressor COP1 specifically interacts with the RING-H2 motif of a novel*Arabidopsis* protein. *J. Biol. Chem.* 274 27674–27681. 10.1074/jbc.274.39.27674 10488108

[B179] Tsuchiya Y. Vidaurre D. Toh S. Hanada A. Nambara E. Kamiya Y. (2010). A small-molecule screen identifies new functions for the plant hormone strigolactone. *Nat. Chem. Biol.* 6 741–749. 10.1038/nchembio.435 20818397

[B180] Uljon S. Xu X. Durzynska I. Stein S. Adelmant G. Marto J. A. (2016). Structural basis for substrate selectivity of the E3 Ligase COP1. *Structure* 24 687–696. 10.1016/j.str.2016.03.002 27041596 PMC4856590

[B181] Vaishak K. P. Yadukrishnan P. Bakshi S. Kushwaha A. K. Ramachandran H. Job N. (2019). The B-box bridge between light and hormones in plants. *J. Photochem. Photobiol. B Biol.* 191 164–174. 10.1016/j.jphotobiol.2018.12.021 30640143

[B182] Vandenbussche F. Habricot Y. Condiff A. S. Maldiney R. Straeten D. V. D. Ahmad M. (2007). HY5 is a point of convergence between cryptochrome and cytokinin signalling pathways in *Arabidopsis thaliana*. *Plant J.* 49 428–441. 10.1111/j.1365-313X.2006.02973.x 17217468

[B183] von Arnim A. G. Deng X. W. (1993). Ring finger motif of *Arabidopsis thaliana* COP1 defines a new class of zinc-binding domain. *J. Biol. Chem.* 268 19626–19631. 10.1016/S0021-9258(19)36562-7 8366106

[B184] von Arnim A. G. Deng X.-W. (1994). Light inactivation of *Arabidopsis* photomorphogenic repressor COP1 involves a cell-specific regulation of its nucleocytoplasmic partitioning. *Cell* 79 1035–1045. 10.1016/0092-8674(94)90034-5 8001131

[B185] von Arnim A. G. Osterlund M. T. Kwok S. F. Deng X. W. (1997). Genetic and developmental control of nuclear accumulation of COP1, a repressor of photomorphogenesis in *Arabidopsis*. *Plant Physiol.* 114 779–788. 10.1104/pp.114.3.779 9232869 PMC158364

[B186] Vu L. D. Xu X. Gevaert K. Smet I. D. (2019). Developmental plasticity at high temperature. *Plant Physiol.* 181 399–411. 10.1104/pp.19.00652 31363006 PMC6776856

[B187] Wang C.-Q. Sarmast M. K. Jiang J. Dehesh K. (2015). The transcriptional regulator BBX19 promotes hypocotyl growth by facilitating COP1-mediated EARLY FLOWERING3 degradation in *Arabidopsis*. *Plant Cell* 27 1128–1139. 10.1105/tpc.15.00044 25841036 PMC4558699

[B188] Wang H. Kang D. Deng X.-W. Wei N. (1999). Evidence for functional conservation of a mammalian homologue of the light-responsive plant protein COP1. *Curr. Biol.* 9 711–S2. 10.1016/S0960-9822(99)80314-5 10395541

[B189] Wang P. Cui X. Zhao C. Shi L. Zhang G. Sun F. (2017). COR27 and COR28 encode nighttime repressors integrating *Arabidopsis* circadian clock and cold response. *J. Integr. Plant Biol.* 59 78–85. 10.1111/jipb.12512 27990760

[B190] Wang W. Chen Q. Botella J. R. Guo S. (2019). Beyond light: insights into the role of constitutively photomorphogenic1 in plant hormonal signaling. *Front. Plant Sci.* 10:557. 10.3389/fpls.2019.00557 31156657 PMC6532413

[B191] Wang W. Paik I. Kim J. Hou X. Sung S. Huq E. (2020). Direct phosphorylation of HY5 by SPA1 kinase to regulate photomorphogenesis in *Arabidopsis*. *bioRxiv* [Preprint]. 10.1101/2020.09.10.291773 PMC8641065 33686674

[B192] Wang Y. Wang Y. Song Z. Zhang H. (2016). Repression of MYBL2 by Both microRNA858a and HY5 leads to the activation of anthocyanin biosynthetic pathway in *Arabidopsis*. *Mol. Plant* 9 1395–1405. 10.1016/j.molp.2016.07.003 27450422

[B193] Wei C.-Q. Chien C.-W. Ai L.-F. Zhao J. Zhang Z. Li K. H. (2016). The *Arabidopsis* B-box protein BZS1/BBX20 interacts with HY5 and mediates strigolactone regulation of photomorphogenesis. *J. Genet. Genom.* 43 555–563. 10.1016/j.jgg.2016.05.007 27523280 PMC5457796

[B194] Weidler G. Oven-Krockhaus S. Z. Heunemann M. Orth C. Schleifenbaum F. Harter K. (2012). Degradation of *Arabidopsis* CRY2 is regulated by SPA proteins and phytochrome A. *Plant Cell* 24 2610–2623. 10.1105/tpc.112.098210 22739826 PMC3406922

[B195] Wertz I. E. O’Rourke K. M. Zhang Z. Dornan D. Arnott D. Deshaies R. J. (2004). Human de-etiolated-1 regulates c-jun by assembling a CUL4A ubiquitin ligase. *Science* 303 1371–1374. 10.1126/science.1093549 14739464

[B196] Xu D. Jiang Y. Li J. Lin F. Holm M. Deng X. W. (2016a). BBX21, an *Arabidopsis* B-box protein, directly activates HY5 and is targeted by COP1 for 26S proteasome-mediated degradation. *PNAS* 113 7655–7660. 10.1073/pnas.1607687113 27325768 PMC4941485

[B197] Xu D. Lin F. Jiang Y. Huang X. Li J. Ling J. (2014). The RING-Finger E3 ubiquitin ligase COP1 SUPPRESSOR1 negatively regulates COP1 abundance in maintaining COP1 homeostasis in dark-grown *Arabidopsis* seedlings. *Plant Cell* 26 1981–1991. 10.1105/tpc.114.124024 24838976 PMC4079363

[B198] Xu D. Lin F. Jiang Y. Ling J. Hettiarachchi C. Tellgren-Roth C. (2015). *Arabidopsis* COP1 SUPPRESSOR 2 represses COP1 E3 ubiquitin ligase activity through their coiled-coil domains association. *PLoS Genet.* 11:e1005747. 10.1371/journal.pgen.1005747 26714275 PMC4694719

[B199] Xu D. Zhu D. Deng X. W. (2016b). The role of COP1 in repression of photoperiodic flowering. *F1000Res.* 5:F1000 Faculty Rev-178. 10.12688/f1000research.7346.1 26949521 PMC4756798

[B200] Xu X. Chi W. Sun X. Feng P. Guo H. Li J. (2016c). Convergence of light and chloroplast signals for de-etiolation through ABI4–HY5 and COP1. *Nat. Plants* 2:16066. 10.1038/nplants.2016.66 27255835

[B201] Xu X. Kathare P. K. Pham V. N. Bu Q. Nguyen A. Huq E. (2017). Reciprocal proteasome-mediated degradation of PIFs and HFR1 underlies photomorphogenic development in *Arabidopsis*. *Development* 144 1831–1840. 10.1242/dev.146936 28420710 PMC5450839

[B202] Xu X. Paik I. Zhu L. Bu Q. Huang X. Deng X. W. (2014). PHYTOCHROME INTERACTING FACTOR1 enhances the E3 ligase activity of CONSTITUTIVE PHOTOMORPHOGENIC1 to synergistically repress photomorphogenesis in *Arabidopsis*. *Plant Cell* 26 1992–2006. 10.1105/tpc.114.125591 24858936 PMC4079364

[B203] Yadav A. Ravindran N. Singh D. Rahul P. V. Datta S. (2020). Role of *Arabidopsis* BBX proteins in light signaling. *J. Plant Biochem. Biotechnol.* 29 623–635. 10.1007/s13562-020-00597-2

[B204] Yadukrishnan P. Datta S. (2020). Light and abscisic acid interplay in early seedling development. *New Phytol.* 229 763–769. 10.1111/nph.16963 32984965

[B205] Yamamoto Y. Y. Deng X.-W. Matsui M. (2001). CIP4, a New COP1 target, is a nucleus-localized positive regulator of *Arabidopsis* photomorphogenesis. *Plant Cell* 13 399–411. 10.1105/tpc.13.2.399 11226193 PMC102250

[B206] Yamamoto Y. Y. Matsui M. Ang L.-H. Deng X.-W. (1998). Role of a COP1 interactive protein in mediating light-regulated gene expression in *Arabidopsis*. *Plant Cell* 10 1083–1094. 10.1105/tpc.10.7.1083 9668129 PMC144059

[B207] Yan H. Marquardt K. Indorf M. Jutt D. Kircher S. Neuhaus G. (2011). Nuclear localization and interaction with COP1 are required for STO/BBX24 function during photomorphogenesis. *Plant Physiol.* 156 1772–1782. 10.1104/pp.111.180208 21685177 PMC3149933

[B208] Yanagawa Y. Sullivan J. A. Komatsu S. Gusmaroli G. Suzuki G. Yin J. (2004). *Arabidopsis* COP10 forms a complex with DDB1 and DET1 in vivo and enhances the activity of ubiquitin conjugating enzymes. *Genes Dev.* 18 2172–2181. 10.1101/gad.1229504 15342494 PMC515294

[B209] Yang J. Lin R. Sullivan J. Hoecker U. Liu B. Xu L. (2005). Light regulates COP1-mediated degradation of HFR1, a transcription factor essential for light signaling in *Arabidopsis*. *Plant Cell* 17 804–821. 10.1105/tpc.104.030205 15705947 PMC1069700

[B210] Yang J. Wang H. (2006). The central coiled-coil domain and carboxyl-terminal WD-repeat domain of *Arabidopsis* SPA1 are responsible for mediating repression of light signaling. *Plant J.* 47 564–576. 10.1111/j.1365-313X.2006.02811.x 16813572

[B211] Yi C. Deng X. W. (2005). COP1 – from plant photomorphogenesis to mammalian tumorigenesis. *Trends Cell Biol.* 15 618–625. 10.1016/j.tcb.2005.09.007 16198569

[B212] Yu J.-W. Rubio V. Lee N.-Y. Bai S. Lee S.-Y. Kim S.-S. (2008). COP1 and ELF3 control circadian function and photoperiodic flowering by regulating GI stability. *Mol. Cell.* 32 617–630. 10.1016/j.molcel.2008.09.026 19061637 PMC2651194

[B213] Yu Y. Huang R. (2017). Integration of ethylene and light signaling affects hypocotyl growth in *Arabidopsis*. *Front. Plant Sci.* 8:57. 10.3389/fpls.2017.00057 28174592 PMC5258764

[B214] Yu Y. Wang J. Zhang Z. Quan R. Zhang H. Deng X. W. (2013). Ethylene promotes hypocotyl growth and HY5 degradation by enhancing the movement of COP1 to the nucleus in the light. *PLoS Genet.* 9:e1004025. 10.1371/journal.pgen.1004025 24348273 PMC3861121

[B215] Yuan T.-T. Xu H. Zhang Q. Zhang L.-Y. Lu Y.-T. (2018). The COP1 target SHI-RELATED SEQUENCE 5 facilitates photomorphogenesis by directly activating photomorphogenesis-promoting genes in *Arabidopsis*. *Plant Cell* 30 2368–2382. 10.1105/tpc.18.00455 30150309 PMC6241259

[B216] Zhao X. Heng Y. Wang X. Deng X. W. Xu D. (2020). A positive feedback loop of BBX11–BBX21–HY5 promotes photomorphogenic development in *Arabidopsis*. *Plant Commun.* 1:e100045. 10.1016/j.xplc.2020.100045 33367254 PMC7747993

[B217] Zhao X. Jiang Y. Li J. Huq E. Chen Z. J. Xu D. (2018). COP1 SUPPRESSOR 4 promotes seedling photomorphogenesis by repressing CCA1 and PIF4 expression in *Arabidopsis*. *PNAS* 115 11631–11636. 10.1073/pnas.1813171115 30352855 PMC6233069

[B218] Zheng N. Schulman B. A. Song L. Miller J. J. Jeffrey P. D. Wang P. (2002). Structure of the Cul1-Rbx1-Skp1-F boxSkp2 SCF ubiquitin ligase complex. *Nature* 416 703–709. 10.1038/416703a 11961546

[B219] Zheng Y. Cui X. Su L. Fang S. Chu J. Gong Q. (2017). Jasmonate inhibits COP1 activity to suppress hypocotyl elongation and promote cotyledon opening in etiolated *Arabidopsis* seedlings. *Plant J.* 90 1144–1155. 10.1111/tpj.13539 28321936

[B220] Zhong S. Shi H. Xue C. Wei N. Guo H. Deng X. W. (2014). Ethylene-orchestrated circuitry coordinates a seedling’s response to soil cover and etiolated growth. *PNAS* 111 3913–3920. 10.1073/pnas.1402491111 24599595 PMC3964075

[B221] Zhou D.-X. Kim Y.-J. Li Y.-F. Carol P. Mache R. (1998). COP1b, an isoform of COP1 generated by alternative splicing, has a negative effect on COP1 function in regulating light-dependent seedling development in *Arabidopsis*. *Mol. Gen. Genet.* 257 387–391. 10.1007/s004380050662 9529519

[B222] Zhou P. Song M. Yang Q. Su L. Hou P. Guo L. (2014). Both PHYTOCHROME RAPIDLY REGULATED1 (PAR1) and PAR2 promote seedling photomorphogenesis in multiple light signaling pathways. *Plant Physiol.* 164 841–852. 10.1104/pp.113.227231 24335334 PMC3912110

[B223] Zhu D. Maier A. Lee J.-H. Laubinger S. Saijo Y. Wang H. (2008). Biochemical characterization of *Arabidopsis* complexes containing constitutively photomorphogenic1 and suppressor of phya proteins in light control of plant development. *Plant Cell* 20 2307–2323. 10.1105/tpc.107.056580 18812498 PMC2570740

[B224] Zhu L. Bu Q. Xu X. Paik I. Huang X. Hoecker U. (2015). CUL4 forms an E3 ligase with COP1 and SPA to promote light-induced degradation of PIF1. *Nat. Commun.* 6:7245. 10.1038/ncomms8245 26037329

[B225] Zhu W. Zhou H. Lin F. Zhao X. Jiang Y. Xu D. (2020). COLD-REGULATED GENE27 integrates signals from light and the circadian clock to promote hypocotyl growth in *Arabidopsis*[OPEN]. *Plant Cell* 32 3155–3169. 10.1105/tpc.20.00192 32732313 PMC7534470

